# Dead and buried? Variation in post-mortem histories revealed through histotaphonomic characterisation of human bone from megalithic graves in Sweden

**DOI:** 10.1371/journal.pone.0204662

**Published:** 2018-10-03

**Authors:** Hege Ingjerd Hollund, Malou Blank, Karl-Göran Sjögren

**Affiliations:** 1 Museum of Archaeology, University of Stavanger, Stavanger, Norway; 2 Department of Historical Studies, University of Gothenburg, Gothenburg, Sweden; Museo delle Civiltà, ITALY

## Abstract

This study investigates possible variation in post-mortem histories during the Neolithic period in southwestern Sweden based on microscopic studies of human bone. Numerous megalithic graves were built in this region and good preservation conditions have left a rich skeletal record. After more than a hundred years of research, it is still a controversy whether or not these skeletal assemblages were the result of primary burials, or ossuaries where skeletonized remains were deposited. In this study we apply histological analysis to obtain insights into post-mortem histories and taphonomic processes affecting the human remains, potentially including funerary rituals. This type of analysis records the condition and traces of degradation found in skeletal material at a microscopic level. Human skeletal material from four different megalithic tombs in the Falbygden area has been sampled and analysed by thin-section light microscopy, and by scanning electron microscopy. The results of the study provide evidence of variation and changes in burial conditions for skeletal remains from the different graves, also for remains from the same grave. Extent of bioerosion varied, from extensive to moderate/arrested, to none. Bone samples from the same graves also differed in the type of staining and mineral inclusions, showing that the non-bioeroded samples relatively early post-mortem must have experienced an anoxic environment, and later a change to an aerated environment. This could be taken as an indication of primary burial somewhere else, but more likely reflect a special micro-environment occurring temporarily in some graves and parts of graves after the tombs were filled with soil and sealed by roof slabs. The study illustrates the usefulness of bone histological analysis in the reconstruction of post-mortem histories, revealing variations not discernible at macro-level that may aid in the interpretations of funerary rituals. However, the results also highlight the issues of equifinality. Based on current data and knowledge, several scenarios are possible. Further histotaphonomic work is advisable, including archaeological remains from megalithic tombs, and bones from taphonomic experiments.

## Introduction

Far from being simply dead and buried, many prehistoric deceased bodies are likely to have gone through a variety of different funerary rituals before becoming part of the archaeological record, most of which will remain unknown to us. The skeletal assemblages of megalithic graves are intriguing examples of this, and the exact nature of mortuary practices carried out in connection with burials in the graves has been the subject of long-lasting debate [1, 2–8]. Still, there is no consensus in how to interpret the remains in these graves. In Scandinavia, the megalithic graves have been described as ossuaries where dismemberment and deposition of defleshed human bodies were practiced [4, 5, 9, 10], while other researchers have suggested that they were predominantly used for primary successive burials [1, 7, 11–14].

**Fig 1:**
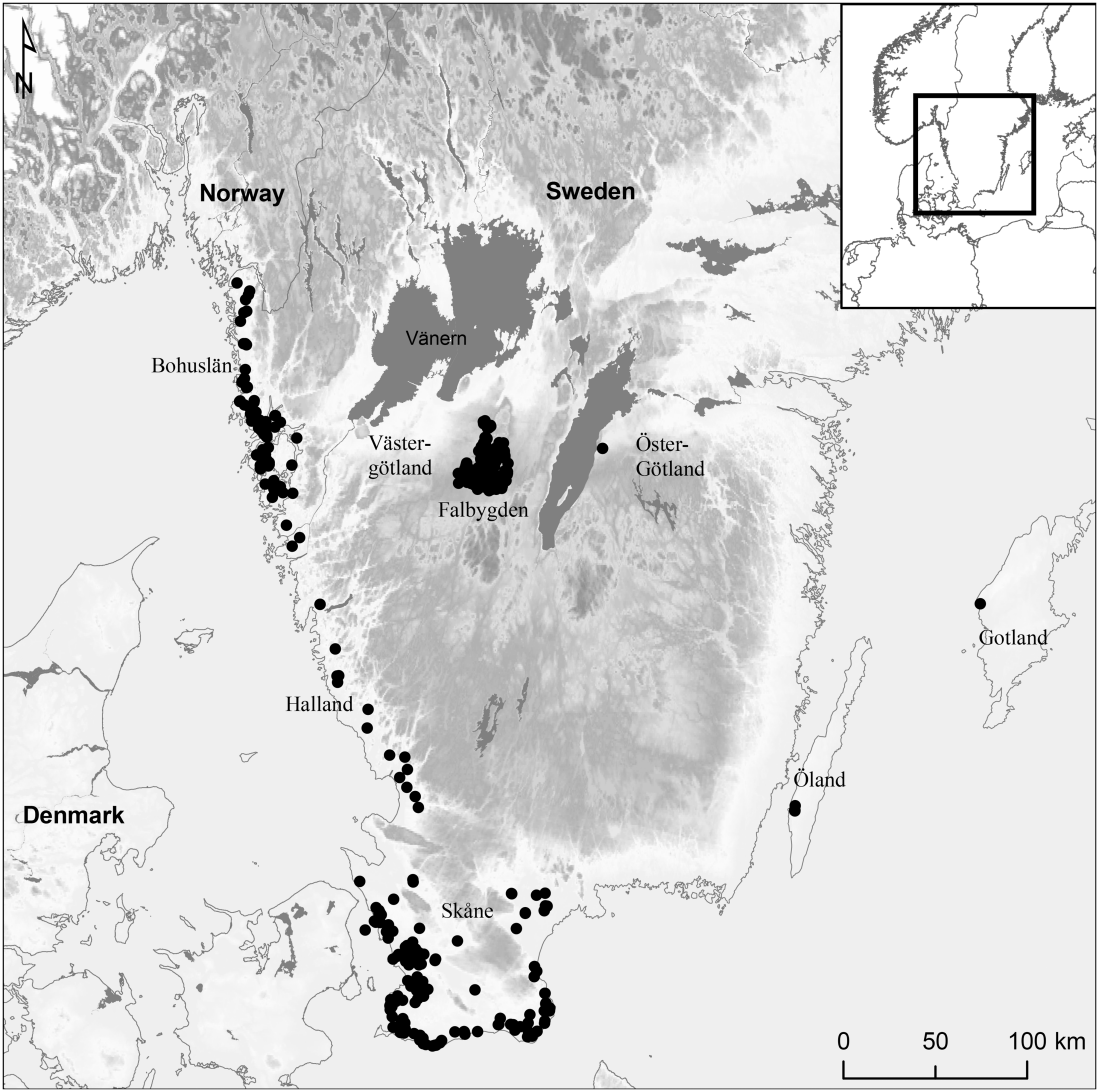
Distribution of passage graves and dolmens in Southern Sweden. Made by K-G. Sjögren, based on Sjögren et al. 2009 [25]. Background map created using data from ESRI. Data and maps licensed to University of Gothenburg.

In this study we investigate the post-mortem histories of dead bodies in Neolithic megalithic graves by histotaphonomic analyses of bone from graves in Falbygden, southwestern Sweden. Scandinavian megalithic graves are divided into three main types: dolmens, passage- and gallery graves. Falbygden, in the inland of southwestern Sweden, is an important area for research on Neolithic megalithic graves as the site of one of Northern Europe’s largest concentrations of passage graves (Fig 1) [9, 12, 15]. The passage graves are relatively homogenous in shape and the predominate type is constructed by a rectangular chamber with a vertically placed passage. The graves are surrounded by mounds which most probably never covered the chambers entirely [12]. The gallery graves in the area are heterogeneous in appearance, although most of them are rectangular and divided in one to three chambers. The chambers may be found above ground or under flat ground and sometimes covered by small mounds or cairns [16, 17]. The dolmens and passage graves seem to have been built over a relatively short time span at the transition between the Early and Middle Neolithic periods, 3300–3000 cal BC, in the cultural setting of the Funnel Beaker Culture [12, 18]. In the same area, numerous gallery graves have also been identified [13, 19]. These graves are mainly assigned to the Late Neolithic (2350–1700 cal BC), even though several have been used into the early Bronze Age and recent work suggest some of the earliest examples were built during the Middle Neolithic [19, 20]. The many megalithic tombs and exceptional preservation of skeletal material, provides an ideal case for investigating synchronic and diachronic variations in burial traditions based on human bones. The Middle Neolithic passage graves have been the subject of systematic studies since the 19^th^ century [12, 21, 22], but less is known about the Late Neolithic gallery graves. The overall picture is that the human bone assemblages appear rather similar in the Late and Middle Neolithic graves. In both passage- and gallery graves a variety of articulated positions are found as well as partially articulated and disarticulated skeletons, some burnt bone and a few rare cases of assemblages of certain bone elements such as skulls [1, 12, 14, 16, 23, 24]. Skeletal remains in the graves are often commingled. Furthermore, many graves were excavated a long time ago, some as early as the 19^th^ and early 20^th^ century, which means that information on exact location and position of bones and skeletons is not always available. These circumstances complicate interpretations of mortuary practices in connection with burials in the megalithic graves.

Histotaphonomy is the study of taphonomic processes at the microstructural scale [26]. Among other things, histological analysis records and semi-quantifies the extent of bioerosion, characteristic diagenetic changes thought to be caused by microorganisms, mainly bacteria. Bioerosion is the most common form of microscopic diagenetic change observed in archaeological bone [27–29]. For a long time in the history of histotaphonomy, there was consensus among researchers that this was a post-depositional phenomenon, caused by soil microorganism after the remains were skeletonised [26]. Since the 1990s several studies have challenged this notion, providing indications that endogenous gut bacteria may be involved in the process [27, 30–33]. If this is correct, it has implications for the reconstruction of early post-mortem histories. British researchers have gone so far as to say that lack of bioerosion is an indication of formerly mummified remains in the archaeological skeletal record [34, 35]. The question of the origin of bone bioeroding microorganisms remains unresolved, however, and may prove difficult to disentangle although actualistic experiments and combinations of bone histological and bacterial DNA (metagenomics) studies may provide answers in the future [26, 28]. The skeletal assemblages from Falbygden provides numerous useful case-studies to explore diagenetic patterns and any correlations with early post-mortem history.

**Fig 2:**
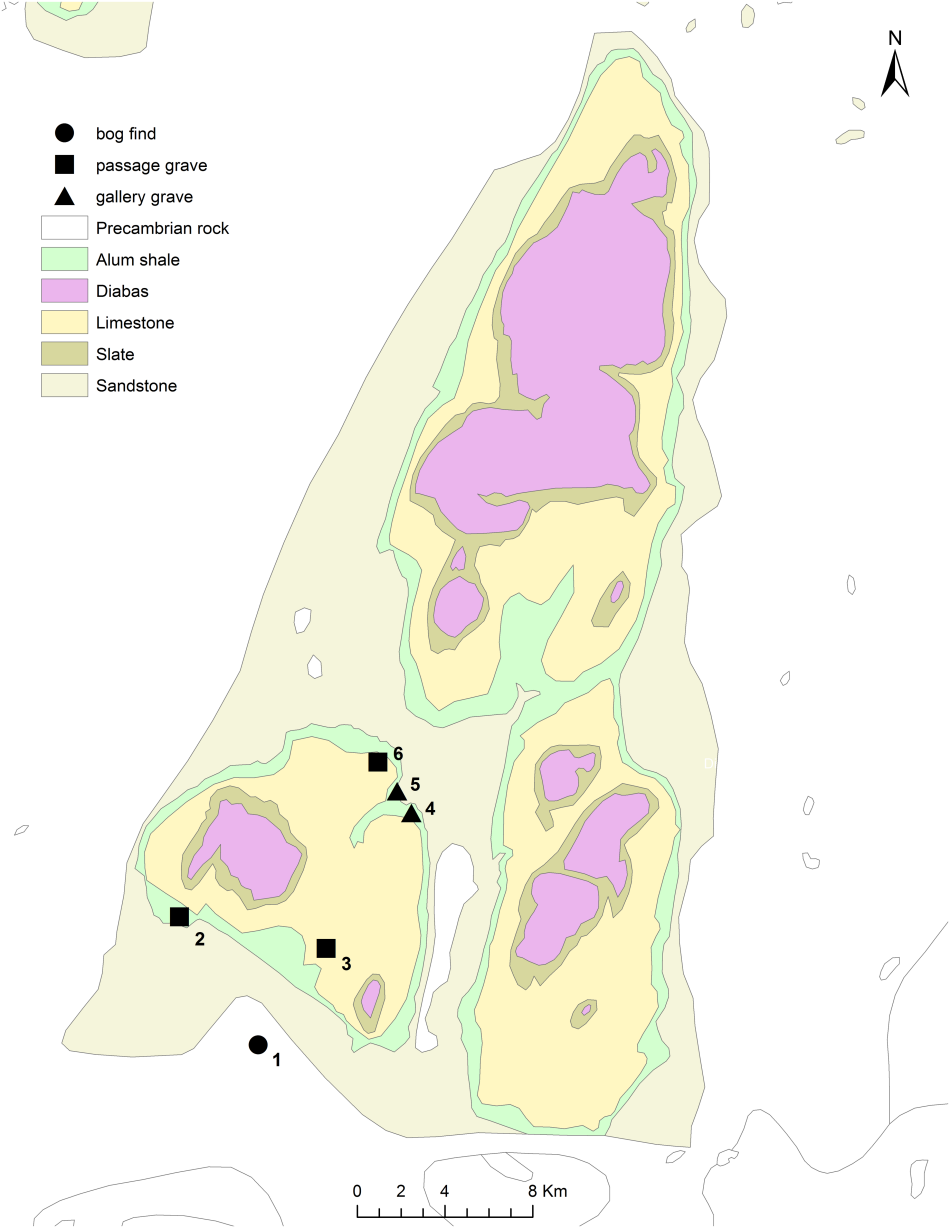
Sites in Falbygden discussed in the article. Map made by Malou Blank in ArchGis 10.1. 1: Rogestorps mosse, 2: Gökhem 94, 3: Falköping östra 1, 4: Torbjörntorp 31, 5: Torbjörntorp 18, 6: Valtorp 2 (Rössberga).

An early study of bone diagenesis included histological analysis of human bone from the Middle Neolithic passage grave Rössberga in Falbygden (Fig 2), which showed that microstructural preservation varied. In this case, the aim was not to reconstruct funerary treatments, but it is worth noting that the analysed assemblage of seventeen bones varied from well preserved to severely bioeroded [36]. In a more recent study by K-G. Sjögren, histological analysis of bone revealed bioerosion in all samples from skeletons buried in the Middle Neolithic Frälsegården passage grave (Gökhem 94:1, Fig 2), ranging from medium to poor preservation (Supporting information, S1 Table). The variation in extent of bioerosion correlated with the level of skeletal disarticulation, and with the different phases of use of the grave. Together with other evidence this diachronic variation was tentatively interpreted as representing a change in funerary ritual [6]. In this study we would like to expand on this work and include skeletons from the Late Neolithic to investigate whether or not similar variation is observed and explore the relationship between diagenetic patterns and other factors such as time period, type of grave, placement and level of skeletal disarticulation. The aim is to reconstruct post-mortem histories and assess to what degree diagenetic characteristics observed reflect diachronic and synchronic variation in funerary treatments.

## Materials and methods

**Fig 3:**
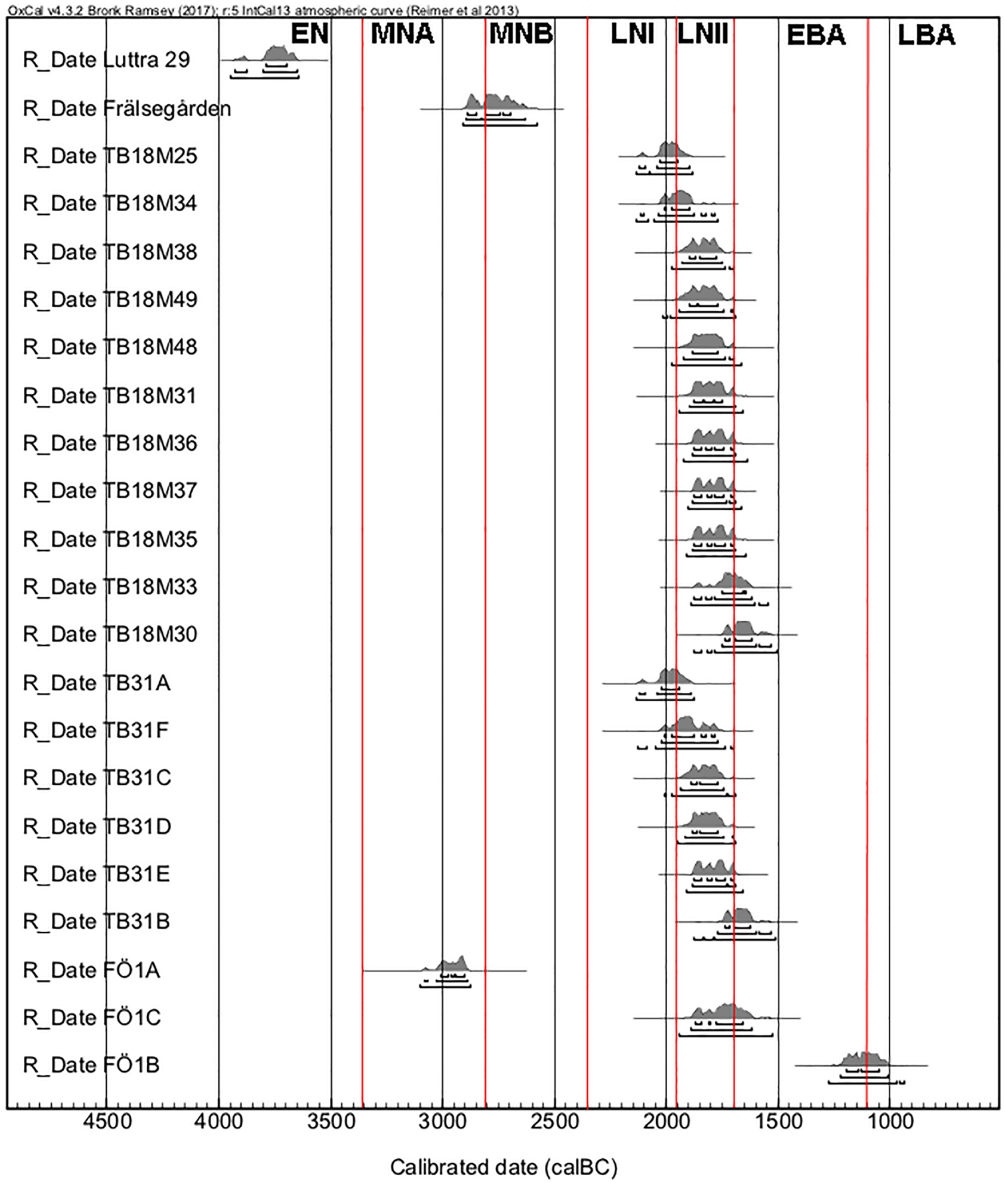
^14^C dates of the 21 sampled individuals. OxCal v.4.2.4 Bronk Ramsey (2013); r:5 IntCal13 atmospheric curve [37]. Red lines mark the different periods: EN = Early Neolithic, MNA = Middle Neolithic A, MNB = Middle Neolithic B, LNI = Late Neolithic I, LNII = Late Neolithic II and EBA = Early Bronze Age.

A total of 21 individuals from four Falbygden graves were radiocarbon dated and sampled for histological analysis. The aim of the sampling strategy was to include individuals from different parts and levels of the graves, and different time periods, although the majority date to the Late Neolithic II period. The material originates from two Middle Neolithic passage graves, Falköping Östra 1 (FÖ1) and Gökhem 94 (GH94:1), and two Late Neolithic gallery graves, Torbjörntorp 18 (TB18), and 31 (TB31) (Figs 2 and 3, Table 1). In addition, the mandible of a wild cat from TB18 was also sampled. Further details on the graves, sites and skeletal assemblages may be found in Table 1 and in the supporting information (S1 File and S2 Table).

**Table 1:** Description of samples.

Grave	Sample id	Placement^a^	Anatomy	Period^b^	Date BP	Date cal BC (95,4%)^c^	C:N	Description^d^
**GH94:1, passage grave**	TSGH94A	Plough layer	Femur	MNB	UBA-29929: 4188±45	2896–2631	3.17	Adult.
**FÖ1, passage grave**	TSFÖ1A	North western corner of the chamber	Femur	MNA	UBA-34280: 4332±39	3083–2889	3.19	Articulated, contracted position. Female, subadult.
	FÖ1B	Mound surrounding chamber	Femur	BAPIV	UBA-34281: 2921±36	1221–1010	3.17	Disturbed. Male, adult.
	FÖ1C	In the rebuilt passage	Ulna	LNII/BAPI	UBA-34282: 3428±55	1887–1616	3.15	Partially articulated, contracted position. Adult.
**TB18, gallery grave**	M25	H4, western slab	Femur	LNI/LNII	UBA-25679: 3624±29	2120–1898		Burnt at moderate temperature (c. 500°C), with soft tissue.
	M30	S4, south western corner	Femur, left	LNII/BAPI	UBA-25756: 3369±40	1751–1533	3.45	Adult.
	M31	H8, by north eastern slab	Femur, left	LNII	UBA-25757: 3479±38	1896–1692	3.16	Adult.
	M33	S24, northern part, middle	Femur, left	LNII/BAPI	UBA-25759: 3412±39	1876–1619	3.21	Adult.
	M34	Uncertain, probably middle of chamber	Femur, left	LNI/LNII	UBA-25760: 3590±36	2112–1782	3.17	Adult.
	M35	Uncertain, probably middle of chamber	Femur, left	LNII	UBA-25761: 3461±32	1882–1692	3.20	Adult.
	M36	H10-11, by south eastern slabs	Femur, left	LNII	UBA-25762: 3467±36	1886–1691	3.21	Adult.
	M37	H7, by northern slab	Femur, left	LNII	UBA-25763: 3464±29	1882–1693	3.22	Adult.
	M38	H4, by western slab	Femur, left	LNII	UBA-25764: 3519±31	1930–1751	3.15	Adult.
	M48	H5, by western slab	Femur, left	LNII	UBA-26012: 3497±39	1925–1696	3.18	Adult.
	M49	S27, by eastern slab	Femur, left	LNII	UBA-26013: 3516±39	1946–1703	3.19	Adult.
	Cat	H4	Mandible					Single fragment of mandible from wild cat.
**TB31, gallery grave**	TB31:A	Lower layer, southern most part	Femur right	LNI/LNII	UBA-34358: 3618±32	2119–1891	3.14	Adult.
	TB31:B	In the entrance, lower body outside the passage	Femur right	LNII/BAPI	UBA-30517: 3379±40	1770–1534	3.18	Articulated, supine position. Female, c. 20 y.
	TB31:C	Southwestern corner	Femur right	LNII	UBA-34595: 3514±35	1935–1746	3.16	Adult.
	TB31:D	Upper layer, northern most part	Femur right	LNII	UBA-34360: 3501±32	1914–1704	3.16	Adult.
	TB31:E	unknown	Femur right	LNII	UBA-34361: 3463±31	1882–1693	3.15	Adult.
	TB31:F	Lower layer, western wall	Femur right	LNI/LNII	UBA-34362: 3561±43	2025–1771	3.15	Adult.

^a^H = slab, S = scull.

^b^ MNB = Middle Neolithic B, MNA = Middle Neolithic A, LNI = Late Neolithic I, LNII = Late Neolithic II, BAPI = Bronze Age period I, BAPVI = Bronze Age period VI.

^c^OxCal v.4.3.2 Bronk Ramsey (2013); r:5IntCal13 atmospheric curve (Reimer et al. 2013).

^d^Adult = >20 years, subadult = <20 years.

See also supporting information (S2 Table) for additional sample information.

Samples of approximately 2x1 centimetres were cut from the diaphysis of a long bone of each individual, preferably femurs, but when this was not available the largest available long bone was chosen. Thin-sections and in some cases additional polished thick-sections were prepared for transmitted light microscopy and scanning electron microscopy respectively. The thin-section preparation involved impregnation with a two-component epoxy and the use of diamond grinding plates to produce thin-sections of 30–50 microns. The thin-sections were studied in the microscope in normal and polarized transmitted light at magnifications of up to x400. Some thin-sections were also studied in the same microscope in reflected normal light. The damage caused by microbes result in localized damage termed microscopical focal destruction (mfd), which have been described by Hackett [38]. The extent of destruction caused by microbial tunneling was assessed qualitatively in the microscope and semi-quantified using the Oxford Histological Index (OHI), developed by Hedges et al. [36] (Table 2).

**Table 2:** The Histological indexes.

OHI/GHI^a^	Approximate % of intact bone	Overall pattern of bioerosion	Description
**0**	< 5	Extensive	No original features identifiable, except that Haversian canals may be identifiable
**1**	<15		Small areas of well-preserved bone present
**2**	<50	Arrested	Some well-preserved bone present between bioeroded/destroyed areas
**3**	>50		Larger areas of well-preserved bone present
**4**	>85		Bone is fairly well preserved with minor amounts of bioeroded/destroyed areas.
**5**	>95	None/Negligible	Very well preserved, similar to modern bone

^a^The Oxford Histological Index (OHI) describes the amount of well-preserved bone not affected by bioerosion [39]. The General Histological Index (GHI) is analogous to OHI, but includes other diagenetic alterations (cracking, staining, generalized destruction) that may also obliterate the bone microstructure.

First, the thin-sections were roughly divided into three regions: periosteal, middle and endosteal, and an OHI-value was given for each region. The final OHI-score is an average of these values. The extent of microcracking was determined using the cracking index (CI) developed by Jans [40], which is the percentage cracked osteons (bone microanatomic units) in one field of view in the microscope. This does not include large cracks as these may be caused by sample preparation. Here, the calculation of the cracking index was an average based on counting in five different fields of view across each thin-section, at magnification ×100. In order to quantify the overall level of preservation, the general histological index (GHI) was applied, following Hollund et al. [41]. The OHI only considers the bone not affected by microbial decay (in the form of clearly identifiable mfds), but these areas may have been affected by other processes. The GHI records the total unaltered microstructure assessing the effect of both bioerosion and other degradation processes such as generalized destruction, staining and extensive cracking (Table 2). Generalized destruction is characterized by general loss of recognizable microstructural features such as the bone lamellar structure, osteocyte lacunae (pores for bone cells) and canaliculi (microscopic canals between osteocytes and vascular canals), and the disintegration, disaggregation and dissociation of osteons [42, 43]. The GHI value is only lowered if the alterations are obstructing the identification and study of the bone micro-anatomy, i.e. that microstructural features such as bone lamellae, canaliculi and osteocyte lacunae have been obliterated.

For five samples, a leftover of the resin-embedded bone used for making a thin-section was polished and coated with carbon for investigation in a scanning electron microscope (SEM). The instrument used was a Zeiss Supra 35 VP SEM with an energy dispersive spectrometer attached (SEM-EDX). This allowed for both back scatter electron (BSE) microscopy and elemental analysis of materials and features observed at high magnification.

## Results

The results of the histological analysis are summarized in Table 3.

**Table 3:** Results of the histological analysis.

Sample id	OHI	GHI	CI^a^	Inclusions	Pattern of bioerosion	SEM-EDX results
**GH94A F74**	0	0	n/a	Orange/reddish-brown grainy material in some Haversian canals; Possible fungal remains.	Extensive	
**FÖ1A**	0	0	n/a		Extensive	
**FÖ1B**	0	0	n/a		Extensive	
**FÖ1C**	5	4	30	Reddish-brown/opaque material filling or lining Haversian canals, cracks and osteocyte lacunae Possible fungal remainswithin Haversian canals.	None	
**M25**	5	4	20	Reddish-brown/opaque material filling or lining Haversian canals, cracks and osteocyte lacunae; Possible fungal remains.within Haversian canals.	None	Manganese
**M30**	5	3	30		None	
**M31**	5	5	80		None	Manganese
**M33**	5	5	50		None	
**M34**	5	4	48		None	
**M35**	3	2	20		Arrested	Manganese
**M36**	5	4	18		None	Manganese
**M37**	3	2	27		Arrested	
**M38**	5	4	27		None	
**M48**	3	3	21		Arrested	
**M49**	5	5	34		None	
**Cat**	2	1	n/a		Arrested	
**TB31:A**	0	0	n/a	Orange/reddish-brown grainy material in some Haversian canals; Possible fungal remains. within Haversian cnaals.	Extensive	
**TB31:B**	0	0	n/a		Extensive	
**TB31:C**	2	1	n/a		Extensive	
**TB31:D**	2	1	n/a		Extensive	
**TB31:E**	2	1	n/a		Extensive	
**TB31:F**	3	3	17	Reddish-brown/opaque material filling or lining Haversian canals, cracks and osteocyte lacunae; Possible fungal remains.within Haversian canals	Arrested	

^a^CI = Cracking Index. In samples with an OHI of 0 the microanatomy is destroyed thus calculations of CI cannot be made.

### Bioerosion

**Fig 4:**
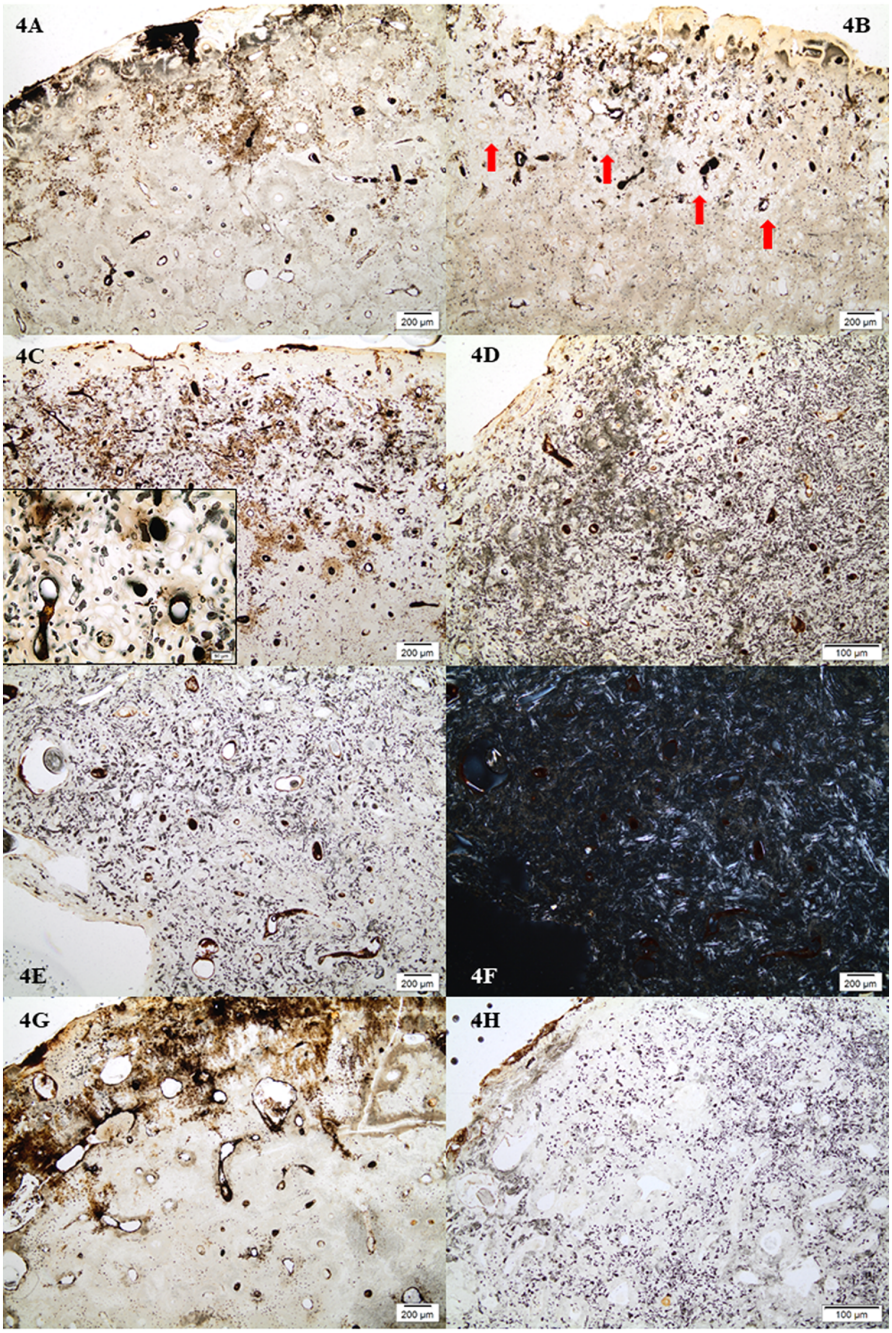
Micrographs showing the patterns of bioerosion and associated staining/inclusions observed. 4A) Sample from TB18 with no bioerosion (M49); 4B) Sample from TB18 with arrested bioerosion (M37). The densest bioerosion is seen in an area close to the periosteal surface, here seen as white spots which are holes left after etching of the localized areas of microbial destruction. Red arrows indicate areas of severe bioerosion; 4C) Sample from TB31 with arrested bioerosion (TB31F). The insert shows the bioerosion at higher magnification where it is possible to discern mfds that are etched and empty, and others that are filled with minerals; 4D) Sample from TB31 with extensive bioerosion (TB31A); 4E) Samples from TB31 with OHI of 2, but with an overall appearance of an extensively bioeroded bone (TB31D); 4F) as 4E but in polarized transmitted light. The bright birefringent areas are the pockets of well-preserved bone; 4G) Sample FÖ1C with no bioerosion; 4H) Sample FÖ1A with extensive bioerosion.

The majority of samples from TB18 displayed no bioerosion with an OHI value of 5 (Fig 4A). Four samples displayed bioerosion with OHI values of 2 or 3, which includes the sample of a wild cat jaw bone. In these cases the densest bioerosion was occurring in an area relatively close to the periosteal surface whereas the periosteal and endosteal areas remained mostly unaffected (Fig 4B). The six samples from TB31 all displayed bioerosion with OHI values of 0 to 3, that is, a pattern of bioerosion that is either extensive (OHI = 0–1), or arrested (OHI = 2–4) (Fig 4C and 4D). The three samples with an OHI value of 2 were, however, in the lower end of the percentage range for this value (15–50% well preserved bone) and appeared extensively bioeroded with dense attack of bioerosion across the whole depth of the section. Many small pockets of well-preserved bone, best visible in transmitted polarized light, were scattered across the whole section giving an OHI of 2 (Fig 4E and 4F). Sample TB31F with an OHI value of 3 had a completely different appearance with an area of dense bioerosion close to the periosteal surface (Fig 4C), similar to the bioeroded samples from TB18. The three samples from FÖ1 showed both extremes. The middle Neolithic teenager from the chamber (FÖ1A), and the Bronze Age individual from the surrounding mound (FÖ1B) were both extensively bioeroded with an OHI of 0, whereas the late Neolithic individual from the passage was well preserved displaying no bioerosion (Fig 4G and 4H). Overall, most samples with an OHI value of 0 would also retain small areas of well-preserved bone, but this would comprise less than 5% of the section.

### Generalized destruction/chemical decay

**Fig 5:**
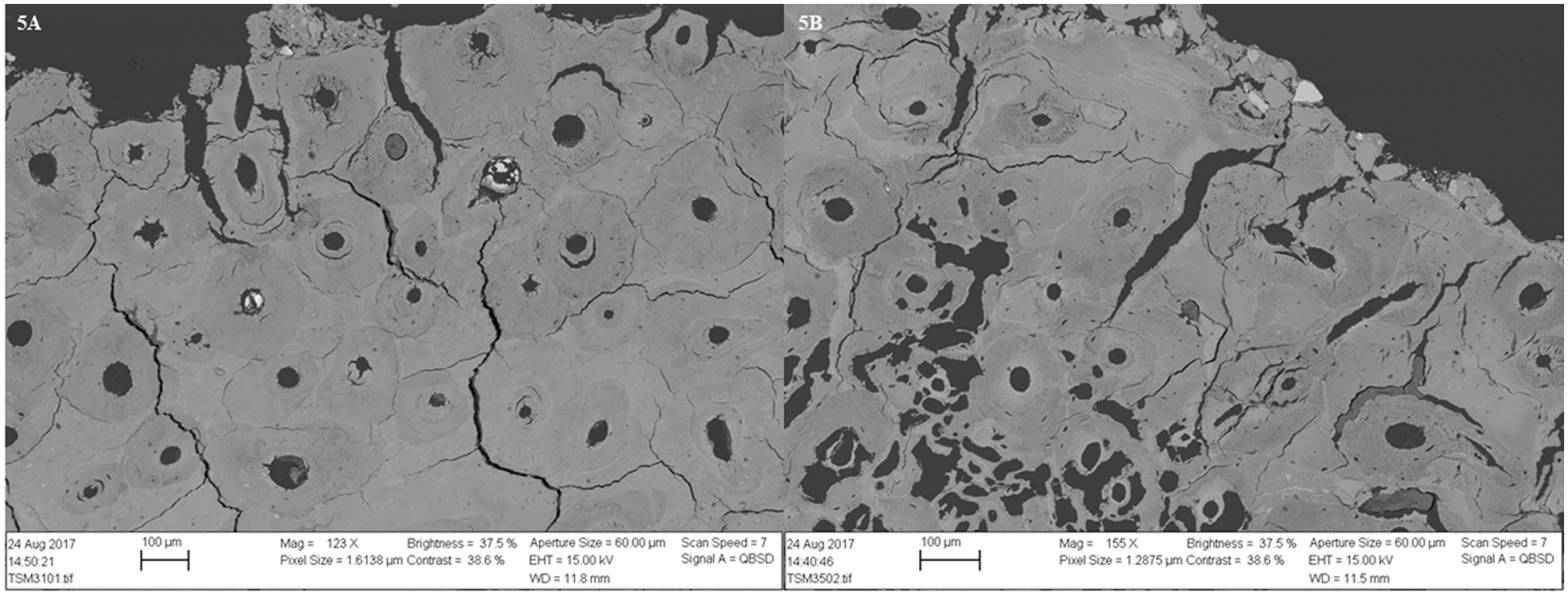
SEM-BSE images of two samples from TB18 showing demineralisation and cracking in the periosteal area. 5A) M31, a sample with no bioerosion; 5B) M35, a sample with a pattern of arrested bioerosion. In the latter image, etched mfds appear as dark irregular holes.

Most samples with no- or arrested bioerosion suffered from generalized destruction to some extent, thus the GHI is lower than OHI in several cases. This type of destruction could be observed as areas where anatomical features like bone lamellae and osteocyte lacunae were disappearing, mainly located in bands along the outer surface. These areas would not be bioeroded but often displayed large cracks and parts of the surface were in some cases lost. Samples with arrested bioerosion *and* generalized destruction contained large areas where destruction caused by microbial decay were etched and leached, leaving round holes with only the hypermineralized cuff that usually surrounds the destroyed foci remaining (Figs 4B and 5B). The SEM images also show how the areas affected by generalized destruction have suffered from demineralisation (Fig 5A and 5B). Limited etching causing generalized destruction also occurred in the extensively bioeroded samples from TB31, FÖ1 and GH94, but generally not to the same extent as in the samples displaying arrested- or no bioerosion. An exception was TB31 E, which displayed etching in larger areas. Overall, the etching was most severe in the samples from TB18.

### Inclusions

**Fig 6:**
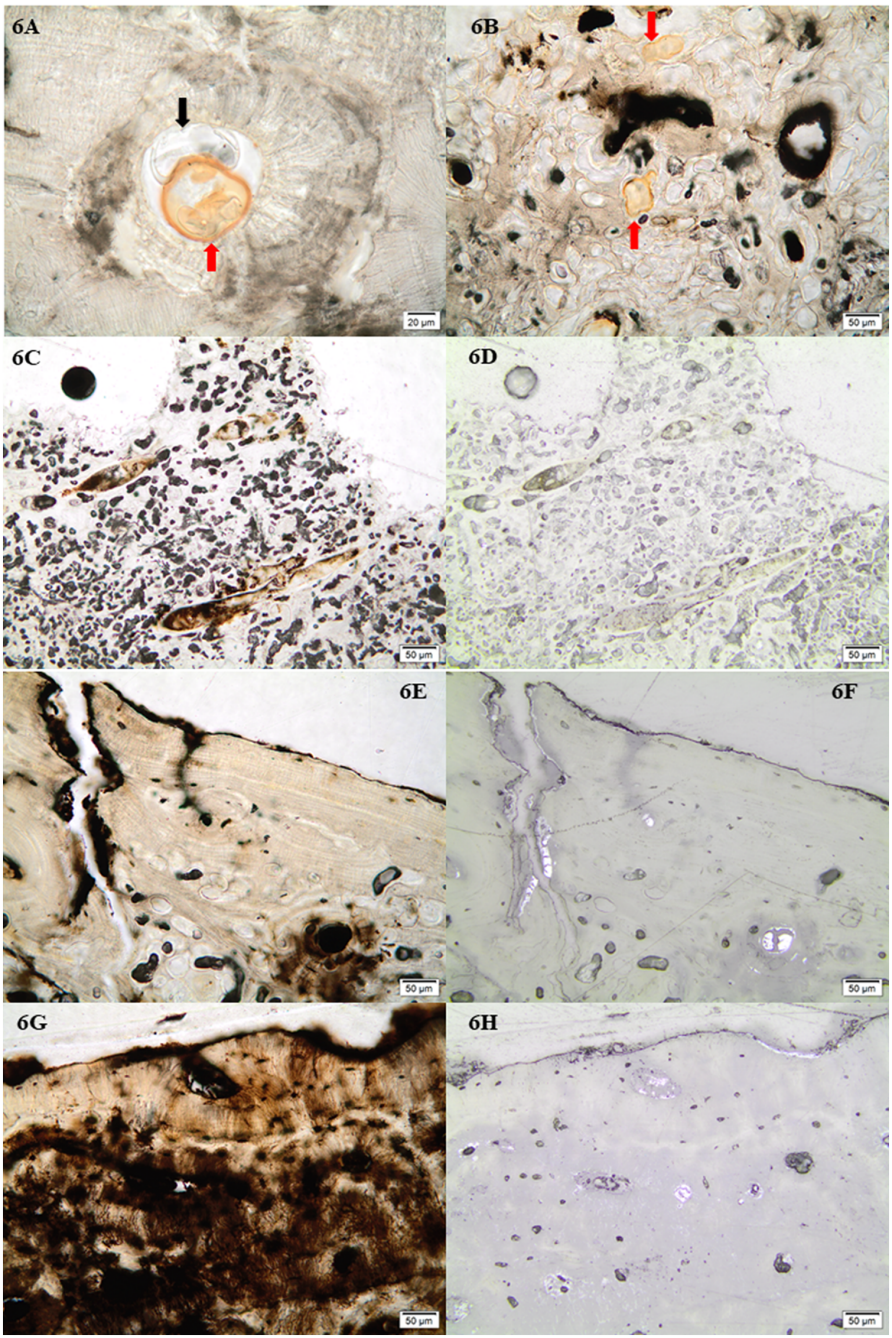
Micrographs showing the inclusions observed. 6A) Possible stained (red arrow) and unstained (black arrow) fungal remains within Haversian canals (M36). Note also the enlarged canaliculi in the form of a striated pattern radiating from the Haversian canal; 6B) Possible stained fungal remains within etched mfds (M37), indicated by red arrows; 6C) Example of grainy reddish-brown inclusions within Haversian canals of extensively bioeroded samples (TB31B); 6D) As 6C, but in bright-field reflected light; 6E) Example from TB31 of dark/opaque inclusions filling Haversian canals, osteocyte lacunae and canaliculi in samples with arrested- and no bioerosion (TB31F); 6F) As 6E, but in bright-field reflected light; 8.7) Example from FÖ1 of dark/opaque inclusions filling Haversian canals, osteocyte lacunae and canaliculi (FÖ1C); 6H) As 6G, but in bright-field reflected light.

**Fig 7:**
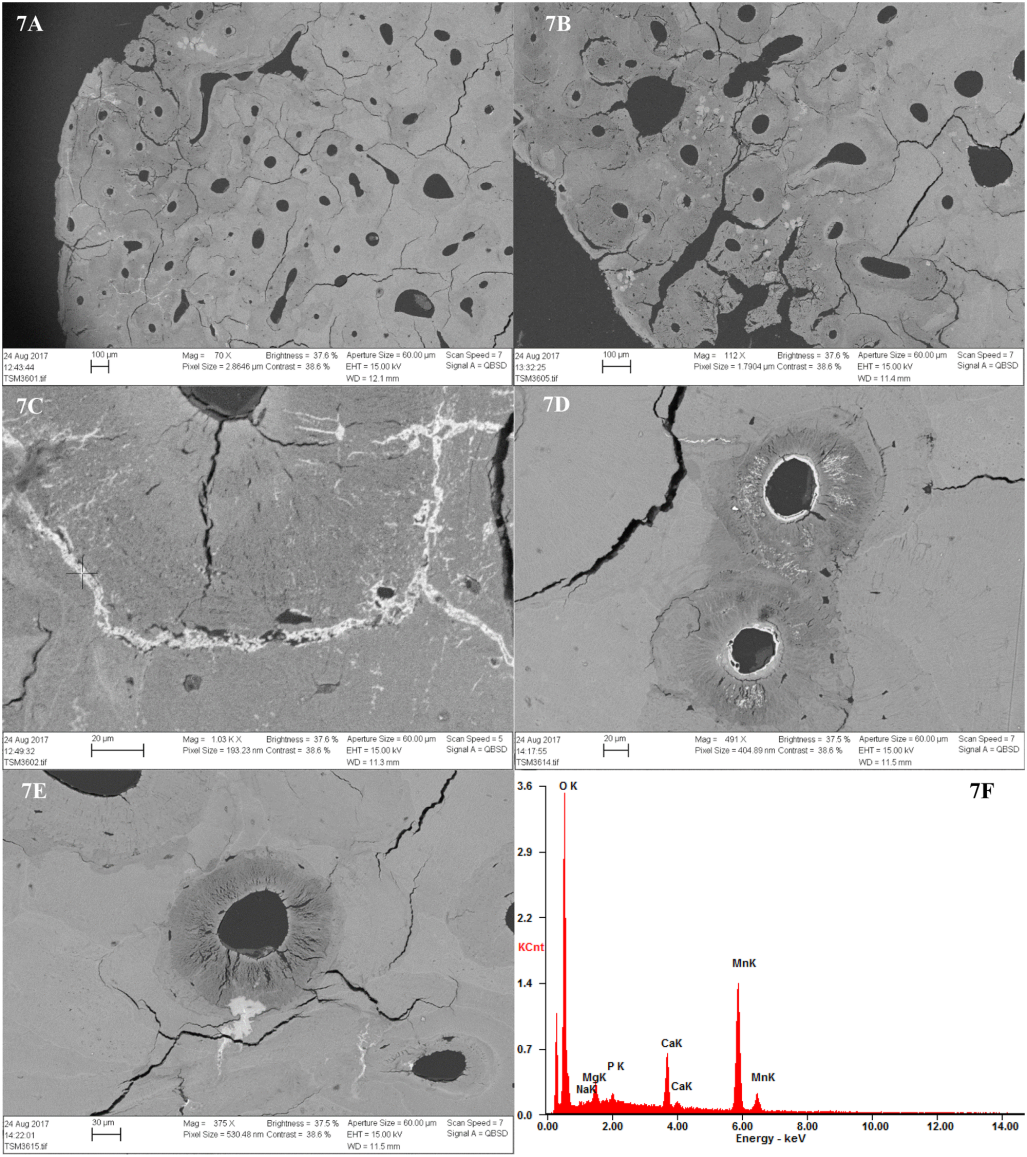
SEM-BSE images and SEM-EDX elemental profiling of dark stains and inclusions observed. 7A-7B) SEM-BSE image of M36 (no bioerosion) showing bright stains and inclusions within cracks and canals. The lighter grey and white colour is evidence of higher density; 7C) Detail of infilled crack. The cross shows the spot where one of the chemical analyses where carried out; 7D-7E) Further details of manganese stains and inclusions within sample M36. Note also the demineralised bone, cracks and stained/enlarged canaliculi, filled with manganese. 7F) SEM spectrum showing the result of the analysis. Manganese (Mn) was detected.

**Fig 8:**
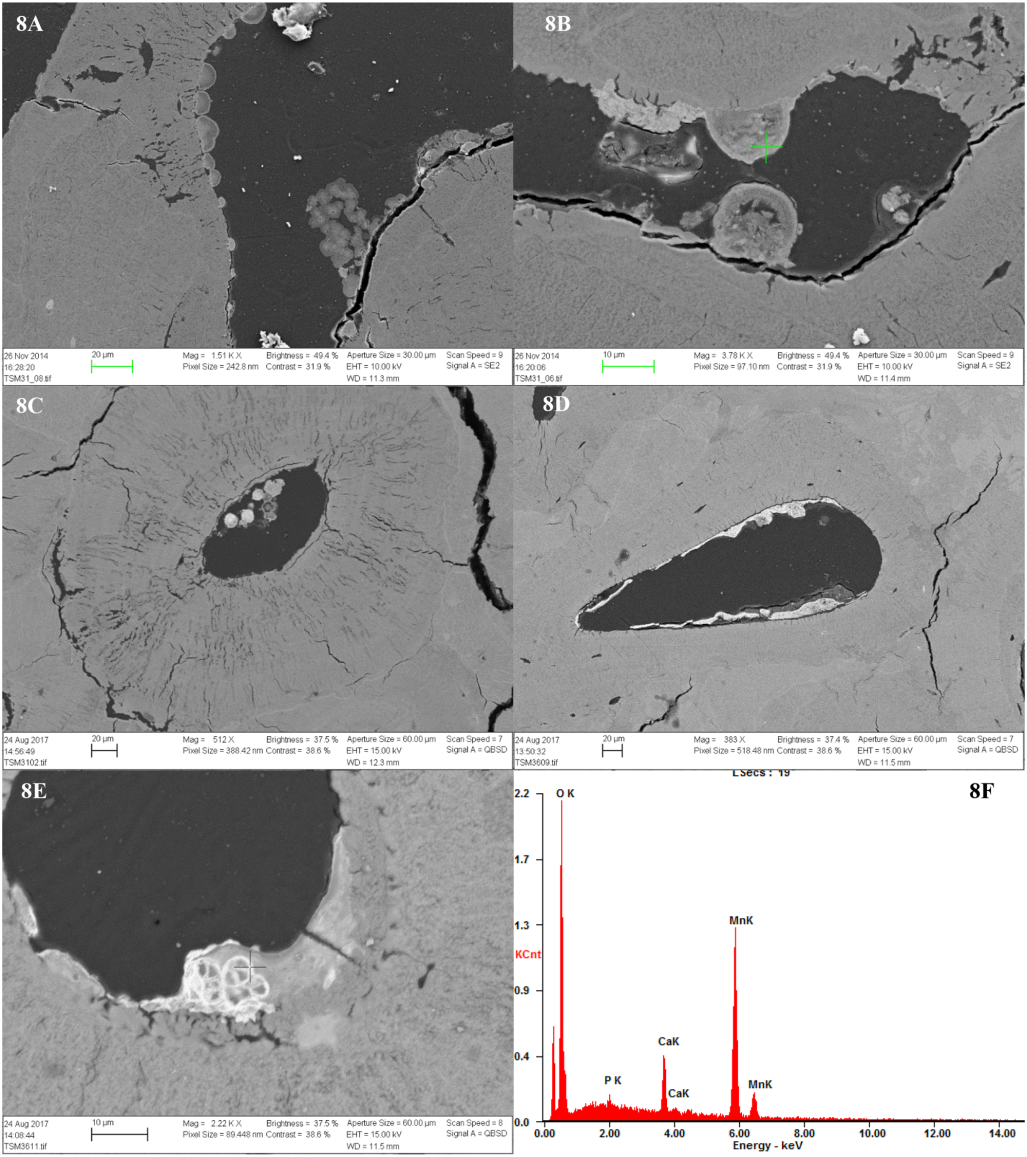
SEM images of globular inclusions. 8A) Globular shapes within Haversian canal in M31; 8B) As 8A, but detail, the cross showing analysed spot; 8C) Globular shapes within Haversian canal in M31. Note also the demineralisation and enlarged canaliculi of the surrounding bone; 8D) Globular shapes within Haversian canal in M36; 8E) Detail of globular shape showing fine walls/filaments with manganese precipitates, cross marks spot analysed; 8F) Results of SEM-EDX analysis of inclusion seen in 8E confirming that they contain manganese.

All samples contained inclusions within Haversian canals, osteocyte lacuna and canaliculi in the form of minerals and possible fungal remains. In the bioeroded and etched samples, inclusions were also observed within the etched microbial focal destructions (mfds) (Figs 4C and 6B). In the light microscope as seen in Fig 6A and 6B, the possible fungal remains are stained globular shapes. The orange colour suggest precipitation of minerals within cell walls. The size and shape seems partially determined/limited by the cavity within which they appear. The mineral inclusions appeared different in the extensively bioeroded samples, compared to the samples with arrested- or no bioerosion. The former contained orange, grainy material within some Haversian canals (Fig 6C and 6D). In the better preserved samples, dark inclusive materials seemed to be filling or lining Haversian canals, and completely filling canaliculi, cracks and osteocyte lacunae, mainly in the area close to the periosteal surface. In bright-field reflected light the dark infills appeared as bright bluish white, massive and angular inclusions (Fig 6E–6H). Chemical analyses by SEM-EDX allowed elemental profiling of stains and inclusions observed in four of the samples from TB18 (M25, M31, M35, M36). These confirmed that the dark stains and inclusions observed in the light microscope are manganese compounds, likely manganese oxides (Figs 5A, 7A–7D and 8D). Furthermore, globular shapes were shown to consist mainly of manganese compounds (Fig 8A–8F). These shapes may be fungal fruiting bodies or other fossilised microbial remains [44–46], and/or the oxidized remains of manganese sulphide grains whose origins are microbially mediated [47]. No fully quantitative measurements were carried out, but in several analysed spots on manganese-containing inclusions, the relative quantity of manganese would amount roughly to between 50 and 70 wt% whereas the remaining elements detected were mainly calcium, oxygen and a few per cent of phosphorus.

### Infiltrations

All samples with no- or arrested bioerosion displayed a similar pattern of staining: The non-bioeroded areas were stained a pale brown whereas there was yellow/orange staining along outer surfaces, and patches of dark reddish brown and black staining, mostly in the etched areas close to the periosteal surface (Fig 4A–4C and 4G). These were visible in SEM-BSE images as brighter stains (Fig 7A and 7B) and chemical spot analyses of these confirmed that they contain manganese. The extensively bioeroded samples displayed no or only limited orange/brown staining along the outer surface (Fig 4D and 4H). Infiltrations and etching generally coincided and in these etched areas there were also stained and enlarged canaliculi. Bones with OHI of 3 and higher all displayed enlarged and/or stained canaliculi (Figs 6A and 8C) in non-bioeroded areas. Enlarged canaliculi is a phenomenon which is still incompletely understood and could be due to either early bacterial action, or chemical decay [38, 41, 48, 49].

### Microcracking

All samples with an OHI above 3 displayed various degrees of microcracking with a cracking index (CI) ranging from ca 20 to 50%. One sample from TB18, M31, was heavily etched across large parts of the sample and displayed both large cracks and extensive microcracking with a CI of 80%. Microcracks were both in the form of cracks radiating from the Haversian canals and following the cementing line around the osteons.

### Heat-induced changes

**Fig 9:**
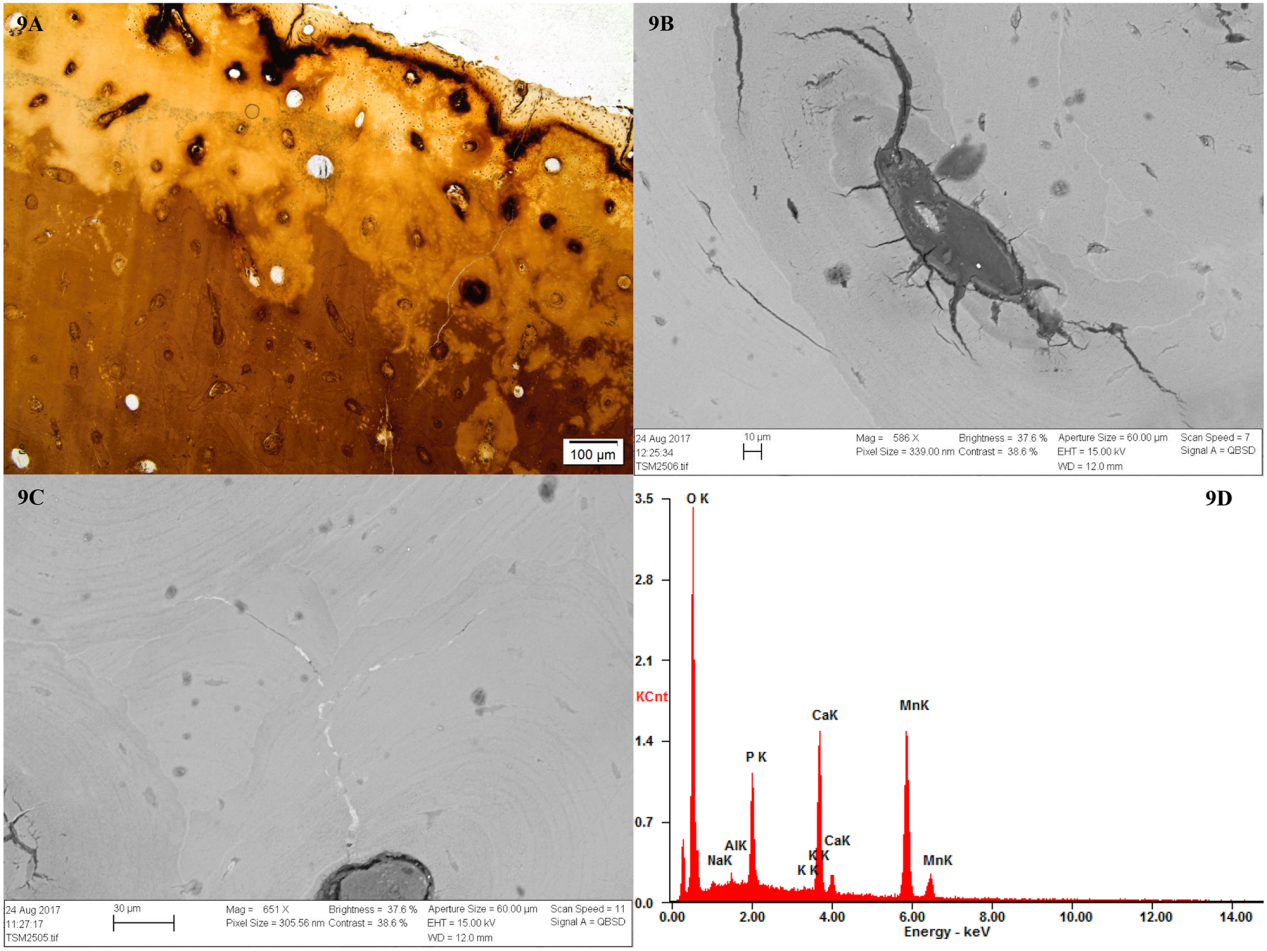
Micrographs and SEM-BSE images showing evidence of heat-induced changes in sample M25. 9A) Overall appearance of the sample with reddish-brown and black discoloration; 9B) SEM-BSE image of Haversian canal showing typical cracking. Note also deposition of carbon in the form of darker grey spots; 9C) SEM-BSE image showing mineral inclusions within cracks, found to contain manganese; 9D) SEM spectrum showing the result of the analysis where manganese (Mn) was detected.

The burnt sample M25 displayed histological alterations caused by low to medium heat intensity (a factor of both temperature and duration) as observed by Hanson and Cain [50]: A dark tan throughout the depth of the sample and reddish-brown and black coloration along the edges, with deposition of carbon along the outer edge and within bone pores. M25 also displayed some of the typical microcracking of burning, with fine cracks that radiated inwards from the surface, and from Haversian canals (Fig 9A–9D).

## Discussion

The results of the histological analysis show that there is variation in diagenetic patterns and degrees of preservation within and between the graves, and across time periods. Samples displayed either extensive or arrested bioerosion, or no bioerosion, and different combinations of these patterns were apparent in the different graves. As the most common faith of archaeological bone is extensive bioerosion, and since a previous histological analysis had shown medium to extensive bioerosion in all samples from GH94:1, it was surprising to observe that the majority of analysed samples from grave TB18, and one from FÖ1, were not bioeroded, A previous study recording bioerosion of bones in the Rössberga tomb [36] showed bones displaying all three patterns (extensive, arrested, no/negligible bioerosion). In our study, we furthermore recorded variation in the extent of microcracking, etching as well as extent and type of staining and inclusions across the analysed assemblage, which showed a relationship with the pattern of bioerosion. The bones displayed one out of three overall diagenetic patterns:

1. Extensive bioerosion, no/limited etching and no manganese precipitates (TB31 FÖ1 and GH94:1).
2. Arrested bioerosion, etching and manganese infiltrations and inclusions (TB31 and TB18).
3. No bioerosion, but etching, manganese infiltrations and inclusions as 2) (TB18 and FÖ1).

The Falbygden tomb assemblages thus display both extremes: no/negligible bioerosion and extensive bioerosion. In the case of Rössberga and FÖ1 both of these patterns are present within the same tomb. Interpreting post-mortem history based on the absence/presence and extent of bone bioerosion is complicated by the unresolved issue of the origin of the organisms involved, and the fact that no experimental work has been carried out to test the occurrence of bone bioerosion in this type of underground chambers. Including data from previous studies of bones from Rössberga [36] and GH94 (Supporting information, S1 Table), the majority of samples so far analysed from Falbygden tombs do display bioerosion (35 out of 48), and about one third are extensively bioeroded. Extensive bioerosion suggest aerated conditions, but when did this bioerosion occur—during putrefaction after being placed in the tombs, or post-burial, after the graves were filled with soil and partially covered by stone slabs—or both? Could it even suggest burial somewhere else before placement in the tombs, as extensive bioerosion is the typical pattern of buried human remains? The previously collected data from GH94:1 seemed to show a relationship between extent of bioerosion and degree of articulation; the articulated remains displayed a pattern of arrested bioerosion, whereas the disarticulated remains were extensively bioeroded (S1 Table). This was interpreted as support of other data suggesting a change in burial practice during the use period of the tomb [6]. The sample from GH94:1 analysed as part of this study also displayed extensive bioerosion, but this was a loose find from the disturbed layer above the better preserved bone layer. There are only a few cases in the current study where we do have some information on location within the grave and level of articulation. Sample TB31:B came from an articulated skeleton which according to the report from 1927 [51] was one of two skeletons lying with the lower part of the body outside of the tomb. In order for this to be kept articulated it must have been one of the last individuals to be placed in the tomb shortly before it was filled and covered by roof slabs and a cairn. This is corroborated by the radiocarbon date (Fig 3). The bone is extensively bioeroded, consistent with decomposition after burial underground of a complete fleshed body immediately post-mortem [52]. Four other individuals from the grave with older radiocarbon dates also displayed extensive bioerosion whereas the final sample from this grave displayed a pattern of arrested bioerosion. In these cases, level of articulation is unknown, but it is likely that the remains lay exposed on the tomb floor for decades or centuries before the grave was filled and closed. Several of the interred remains may for an extended period of time have been isolated from the soil. Grave TB31 had a floor made of limestone slabs, whereas the other tombs had soil floors. In TB31, pig phalanges have also been found, which are frequent finds in the Falbygden graves. Based on their placement in relation to articulated human remains they have been interpreted as the body being wrapped in skins [1], which may also have limited the contact with soil. The bone sample from the articulated and crouched Middle Neolithic individual in the chamber of FÖ1 is also extensively bioeroded, whereas the similarly articulated body from the Late Neolithic in the passage of the same grave displays no bioerosion. There is thus seemingly no relationship between bioerosion and degree of articulation.

It may be argued that placement of a complete body in an underground tomb, with its cooler indoor climate, will offer some protection from insects and scavengers, slowing down the rate of soft tissue loss. This would allow bacteria to effectively spread throughout the whole body and produce extensively bioeroded bones, similarly to what is observed in buried inhumations. In this case, some special early post-mortem treatment or depositional environment would have to be assumed for the non-bioeroded remains. It would also imply that endogenous bacteria are involved in bone bioerosion and/or would necessitate the contact with soil early post-mortem. Forensic work has shown the slowing down of soft tissue decay in indoor or cave environments due to lower insect activity [53–56], but it is uncertain to what degree this would affect processes of bone decay as this has never been tested. It may be suggested that the fact that the tombs remained open and regularly revisited for a long period of time, means that processes are more similar to those of surface-exposed carcasses, although skeletonisation rates may be somewhat slower than above ground. Long-term taphonomic experiments studying diagenetic changes in bone from surface-exposed animal carcasses show that no, or only limited bone bioerosion occur due to fast skeletonisation rates [49, 57]. A study comparing bone collagen preservation in a Roman period catacomb in Italy, to that of open-air burial sites in the same area, found that collagen was overall better preserved in the catacombs [58], which may lend support to the idea that bioerosion is inhibited in underground enclosed spaces. This assumption has been used to interpret arrested bioerosion in British Iron Age skeletons as reflecting deposition in mortuary houses [33]. If we assume that deposition in an underground chamber in itself leads to no- or arrested bioerosion, the extent of bioerosion would be controlled by the local burial environment after the tombs were filled with soil, and factors such as temperature, pH, geohydrological conditions and oxygen availability [28, 29, 52, 59].

The observed variation in inclusions, staining, etching and cracking provide further evidence of the environmental conditions experienced by the three diagenetic groups. Dark staining and inclusions were only present in the samples with arrested- or no bioerosion and these were found by chemical analysis to contain manganese. Manganese is ubiquitous in soils, but the solubilisation of manganese ions and precipitation of black and dark brown insoluble manganese oxides requires circulation of groundwater, with alternating wet anoxic and dry oxic conditions [41, 60, 61]. During a period of wet, anoxic and reducing environment, manganese would be present as ions and could enter the bone via the groundwater [62, 63, 64]. The environment must subsequently have been oxygenated allowing the precipitation of manganese oxides. The infiltration by tannins, humics and mineral precipitates would inhibit further bone bioerosion [65–67]. Both the precipitation of minerals, and the change to drier conditions may then explain the microcracking observed in the stained samples. Fossil bones in aerated karstic caves are often stained by manganese oxides and the origin of the manganese is thought to be circulating water, or the manganese present in the surrounding limestone rock dissolved by groundwater [64, 68, 69]. The limestone slabs used in the tomb construction are thus possible sources of manganese.

The precipitation of manganese oxides may also have been microbially mediated [44]. Certain microorganisms are able to free manganese into the environment by utilizing the organic part of complex molecules [45]. Metals may precipitate onto microbial cells through biologically controlled or induced mineralization and this may occur in a variety of environments with redox gradients. Metabolic processes of manganese-oxidizing bacteria can furthermore produce considerable acidity. Microscopic globular and dome-shaped manganese-precipitates in cave minerals, dinosaur bones, marine sediments and sedimentary rocks have been interpreted as fossilised bacterial or fungal cells [45–47, 70], which is possibly also what was observed in the Falbygden bones (Fig 8). Similarly sized and shaped manganese-oxide spheroids have also been observed in deep-sea sediments, around and within plankton and diatom skeletons possibly mediated by the endogenous bacteria of the plankton and diatom post-mortem [71].

The presence of these stains provide evidence that all bones analysed from TB18, one out of four from TB31 and one of two from FÖ1 have experienced fluctuating redox conditions and at some point experienced a wet, reducing anoxic environment. From the tree diagenetic patterns observed and summarised above we may thus discern three different environmental conditions and sequences of events likely occurring relatively early in the several thousand year post-mortem history of these bones:

1. Extensive bioerosion: Free-draining aerated conditions throughout the post-mortem history.
2. Arrested bioerosion and manganese: A fluctuating environment with an extended period in aerated conditions allowing a certain level of bioerosion, a change to anoxic, reducing and wet conditions, changing again to an oxygenated environment.
3. No bioerosion and manganese: Anoxic, wet and reducing conditions relatively early post-mortem or post-burial, later changing to aerated.

Anoxic, reducing and acidic conditions has thus led to arrested- or no bioerosion in some skeletal remains and subsequent aeration caused precipitation of manganese oxides. As with the bioerosion, the question then becomes: At what point in the post-mortem history did this occur? Hedges [36] describe the burial environment of the Rössberga tomb as …*relatively cool*, *moist*, *fine*, *well-drained soil of approximately neutral pH*, i.e. a type of burial environment in which we would expect extensive bioerosion as indeed is the case in most of the so far analysed material from Falbygden tombs. This may point to a different burial environment within TB18, where all samples display manganese stains, and most are free of bioerosion. This assumes a scenario where bioerosion is inhibited before most remains are skeletonised and embedded in a soil matrix. It is not likely that the burial conditions of the graves under study were vastly different. The soil in Falbygden generally consists of fine-grained (silt-sand) moraine material. The two gallery graves at Torbjörntorp lie somewhat lower than the passage graves which may suggest a wetter environment, but there should be no great difference between TB18 and TB31. The grave fill in TB18 was described, however, as being filled with damp sand, whereas TB31 was described as filled with black soil (see supporting information, S1 File). It was also noted during excavation that bones and artefacts were etched and damaged and that the best preservation of skeletons was found closest to the limestone slabs [72]. The sand in TB18, perhaps of an acidic type, could have constituted a corrosive environment also not beneficial to the bacteria normally involved in the erosion of bone. Studies have found that early taphonomic processes have a great impact on the survival and preservation of bone, but that this effect is most significant in benign soils of neutral pH. In corrosive burial environments the soil acidity has an overriding effect and soil conditions become more important in determining the long-term fate of the material [29, 59]. The variation in preservation conditions observed within TB18 shows how conditions can vary even within a small space. This was also found by the study of bone preservation in a Roman period catacomb in Italy where among other things the size of the chamber affected bone preservation [58]. The fact that the wild cat bone from TB18 displayed the same diagenetic characteristics as the human bones support that this pattern is a result of the tomb environment. Wild cats may have been extinct in Sweden already in the early Bronze Age, which increases the likelihood of this being interred in the grave around the same time as the Neolithic humans [73, 74]. The bone most likely represents the remains of a cat corpse interred with the humans as part of the grave goods. It cannot be excluded, however, that it was used as personal adornment, worn as a pendant or sown onto clothes.

The tombs TB31 and FÖ1 that contain bones both with and without manganese stains, may also attest to quite dramatic variations in local burial conditions. The two skeletons from FÖ1, although dating from two different periods, and found in two different parts of the grave, appear similarly articulated in a crouched position. Since there is some relationship with time period in our data (all non-bioeroded bones so far date to LN and EBA), the contrasting diagenetic patterns may relate to whether or not the remains were interred early, the earlier remains perhaps being covered by soil before starting a new phase of use. There is evidence of stratigraphic phasing in some graves, with up to three bone-containing layers (Supporting information, S1 File). The earlier remains may thus have experienced a more aerated, free-draining environment whereas the later remains were interred and covered when sediment build-up was greater and the amount of decaying organic matter was higher. This may have led to anoxic conditions in the sediments immediately surrounding the remains that still retained organic tissue. As organic matter degrades, a low-oxygen micro-environment is produced, leading to conditions under which iron and manganese minerals will become soluble. The minerals may be redistributed several centimetres away from the breaking-down organic matter [68] and could thus enter the bone where they later precipitate as insoluble manganese oxides. In other, perhaps older areas of the grave, less organic material is present, but a free-draining, and aerated burial environment could have allowed bacterial decay of presumably by then skeletonised remains.

The scenario described above is not possible if we assume that bodies placed in underground tombs would be expected to suffer extensive bone bioerosion by gut and/or soil bacteria, similarly to buried remains. Nor if one assumes that bone bioerosion is driven by gut bacteria, occurring only in connection with soft tissue putrefaction. Recent studies from the British Isles have suggested that the lack or arrest of bone bioerosion in prehistoric times is an indication of systematic and deliberate use of techniques to limit soft tissue putrefaction, with the aim to create corpses with intact soft tissue, i.e. mummies [34, 35, 75, 76]. One of these studies suggested temporary burial in bogs as a method for producing mummies during the Bronze Age in Britain, based partly on a bone diagenetic pattern inconsistent with that of the burial environment in which they were found. Among other things, the bone displayed arrested bioerosion [77]. Burial in waterlogged anoxic conditions, or within reach of a fluctuating water-table, has been shown to produce bone with arrested- or no bioerosion [34, 35, 41, 52, 63, 78, 79]. Bog burial is certainly a method that would have been readily available to the people in Falbygden as the area is rich in fens and bogs, also in close vicinity to the graves [80]. The Neolithic people are likely to have been familiar with the preservative powers of peats and the deposition of both artefacts and humans in the bogs of Falbygden are known from the period [81]. A sample from a well-preserved Early Neolithic bog skeleton from the Rogestorps bog in Falbygden (Figs 2 and 3), the so-called Raspberry girl (Hallonflickan), has recently been analysed by the first author. The Raspberry girl body was deposited in a marsh peat with underlying chalk gyttja, which produced well preserved bones with mineral inclusions in the form of pyrite (iron sulphide), but was evidently not conducive to the survival of soft tissues [81]. No pyrite was found in the entombed bones and if some of the bodies were first deposited in a bog to achieve soft tissue preservation, it would have to be one with a different type of environment to that of the Raspberry girl. According to Fischer [82], the raised bogs provide the best preservation of soft tissue. These bogs are low in oxygen and dominated by the peat moss sphagnum. A breakdown product of this moss has been found to be the essential preserving agent due to good antimicrobial and tanning properties [63, 67]. Few palaeobotanical studies have been carried out in the area, but it is known that thick layers of peat bog developed in Falbygden during the sub-boreal period [83, 84] and it seems that the transition from the development of fens to sphagnum peat bogs occurred during the Neolithic, making it possible that the sphagnum type bogs were present in the Late Neolithic [84]. Experimental burial of piglets in sphagnum peat bogs has shown that a mummy could be produced within half a year, and that wrapping improved preservation [85]. Very few bog bodies and bog skeletons have been analysed histologically, but a couple of studies show that these have suffered very little or no bioerosion [35, 86]. Decalcification is, on the other hand, common in European bog bodies due to the sequestering capacity of compounds in the sphagnum moss causing rapid demineralisation to a certain depth from the periosteal surface [63, 87]. This is similar to the pattern of demineralization observed in many of the analysed bone samples with arrested- and no bioerosion.

Admittedly, mummification by temporary bog burial is an extreme scenario, and aside from the spectacularly well preserved bog bodies and skeletons in Northern Europe, no evidence exists of such practices in connection with funerary rites, prehistoric or historic. The bog bodies were likely deposited without the intention of ever recovering them. The multi-analytical approaches by Smith et al. [76] and Parker Pearson et al. [77, 88] in their studies of Bronze Age burials in the UK provide convincing evidence that in these special cases, retention and circulation of mummified bodies after death did occur although the method of mummification have not been possible to identify. Whether or not such practices were common during prehistoric times in Northern Europe is difficult to prove, as the subsequent burial environment does not normally preserve soft tissue. One of the bones from TB18 may hint at such a practice as it displays evidence at macroscopic level of exposure to moderate heat (< 500 °C), most likely with soft tissue intact, as identified by osteoarchaeologist (Åsa Larsson pers. comm.). We also found evidence of low temperature heating at microscopic level (Fig 9), and the manganese stains evidence of temporary burial in an anoxic environment. Another possible explanation for the observed diagenetic pattern is that the bones with no- or arrested bioerosion originally were mummified, by natural and/or artificial means, and would have retained soft tissue for a longer period even after burial and closure of the tombs, creating the special microclimates and/or microbial activity necessary for the mobilisation of manganese. It cannot be excluded that the tomb environment itself may have had a preservative effect in some cases, perhaps at certain times of the year, allowing natural desiccation in the same way as underground catacombs [89].

## Conclusions

This histotaphonomic investigation of a bone assemblage from Neolithic megalithic graves in southwestern Sweden has revealed both diachronic and synchronic variation in post-mortem histories, based on different diagenetic patterns observed. Dark stains and inclusions of manganese oxides were restricted to bones with no- or arrested bioerosion, showing that these bones had temporarily experienced an anoxic burial environment. Both stained and unstained bones were found in two of the graves, whereas all the bones from the late Neolithic passage grave TB18 were stained, and most were not bioeroded. Possible scenarios to explain this include temporary burial in an anoxic environment such as a peat bog, but is perhaps more likely related to localized and temporary variation in redox potential within and between tombs due to factors such as depth, differential sediments and/or build-up of organic material. Some or all of the manganese precipitates may be microbially mediated, suggesting that the precipitation occurred when soft tissue was still present. This opens up the possibility that the stained bones were somehow mummified, naturally or artificially, and in that way gave rise to special microbial communities and micro-environments within the tombs different to those of presumably skeletonised remains. The extensively bioeroded bones with no manganese precipitates must have experienced an oxygenated environment throughout the post-mortem period. It is uncertain, however, whether or not the extensive bioerosion observed could have occurred during putrefaction in the open tomb, or started/continued after the skeletonised bones were buried.

The bone assemblages from Middle and Late Neolithic graves appear rather similar from a macroscopic perspective. The histological analyses has shown that there are variations both within and between these periods and the current study agree with recent work promoting this method as a powerful additional tool in funerary archaeology. Both this and other case-studies allows us to speculate that in certain cases active measures were taken to promote soft tissue preservation, in our case suggested by a burnt and manganese-stained bone. Exactly what these measures were, and the extent of any such practice, remains unresolved, but future multi-analytical studies including histology may provide some answers. Whereas previous histological studies of funerary rites have focused on the absence/presence of bacterial degradation, the current study shows how also other diagenetic traits observed in the microscope can add to the post-mortem history and strengthen interpretations. Still, the issue of equifinality leaves us with several possible scenarios based on the presented evidence, particularly since the bone eroding microorganisms have not been identified. The case of Falbygden provides a possibility to explore this further due to the large and diverse skeletal collections available and more histological analyses should be carried out on material from secure and well described contexts. In addition there is a real need for more experimental work including the effect on bone histology of burial in above- and underground mortuary houses/chambers. As more osteological and bioarchaeological analyses are being carried out on this material, we will also get a better understanding of the people buried in the megalithic tombs, and which individuals have experienced different post-mortem histories, potentially informing interpretations of funerary treatments and burial traditions.

## Supporting information

S1 Fig Plan of Torbjörntorp 18. Made by Malou Blank in ArchGis 10.1, based on Ullenius [72]. Except for bone associated with skulls S4, S24 and S27 plotted on the drawing, the location of bones were documented by closest slab, numbered 1–20.
(TIF)

S2 Fig Plan of Torbjörntorp 31. Made by Malou Blank in ArchGis 10.1, based on Sahlström [51].
(TIF)

S3 Fig Sample locations in Falköping östra 1. Made by Malou Blank in ArchGis 10.1.
(TIF)

S4 Fig Bone density at the Frälsegården passage grave (Gökhem 94:1). Kernel density plot, calculated from bone midpoints. Made by K-G. Sjögren in ArchGis, reprinted from Sjögren (2008) [90], under a CC BY license, with permission from University of Gothenburg, original copyright.
(TIFF)

S1 File Site descriptions. Detailed descriptions of the four graves and skeletal assemblages discussed in the article.
(DOCX)

S2 File Letter from Åsa Larsson, personal communication. (DOCX)

S1 Table Histological data from previous study on bone from Gökhem 94:1. (DOCX)

S2 Table Additional sample information. (DOCX)
