# Supplementary figures and images for: Dead and buried? Variation in post-mortem histories revealed through histotaphonomic characterisation of human bone from megalithic graves in Sweden
Source: PLoS One. 2018 Oct 3;13(10):e0204662. doi: 10.1371/journal.pone.0204662 (PMC6169911; doi:10.1371/journal.pone.0204662)

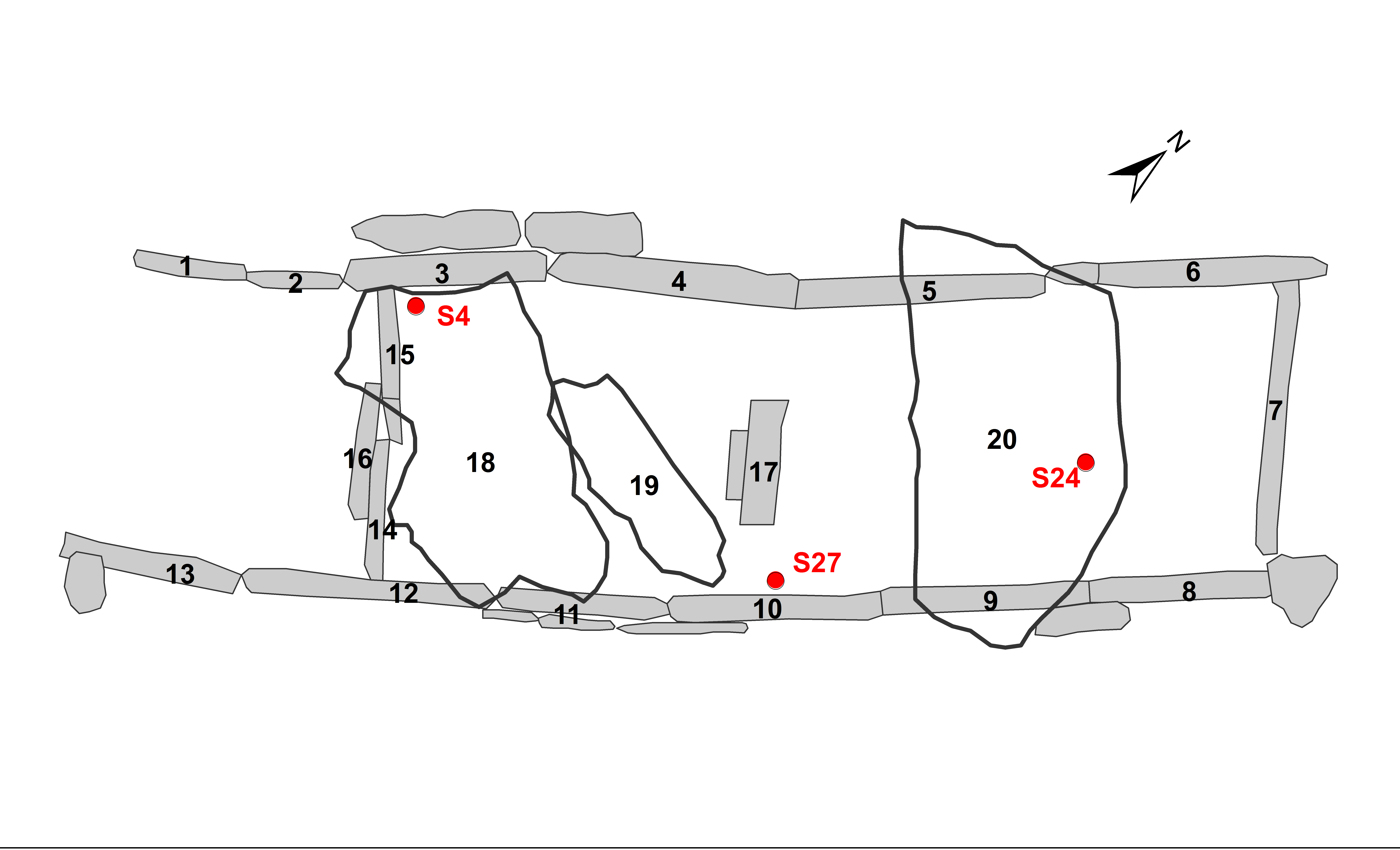

Supplement: S1 Fig — Made by Malou Blank in ArchGis 10.1, based on Ullenius [72]. Except for bone associated with skulls S4, S24 and S27 plotted on the drawing, the location of bones were documented by closest slab, numbered 1–20. (TIF) [file pone.0204662.s001.tif]

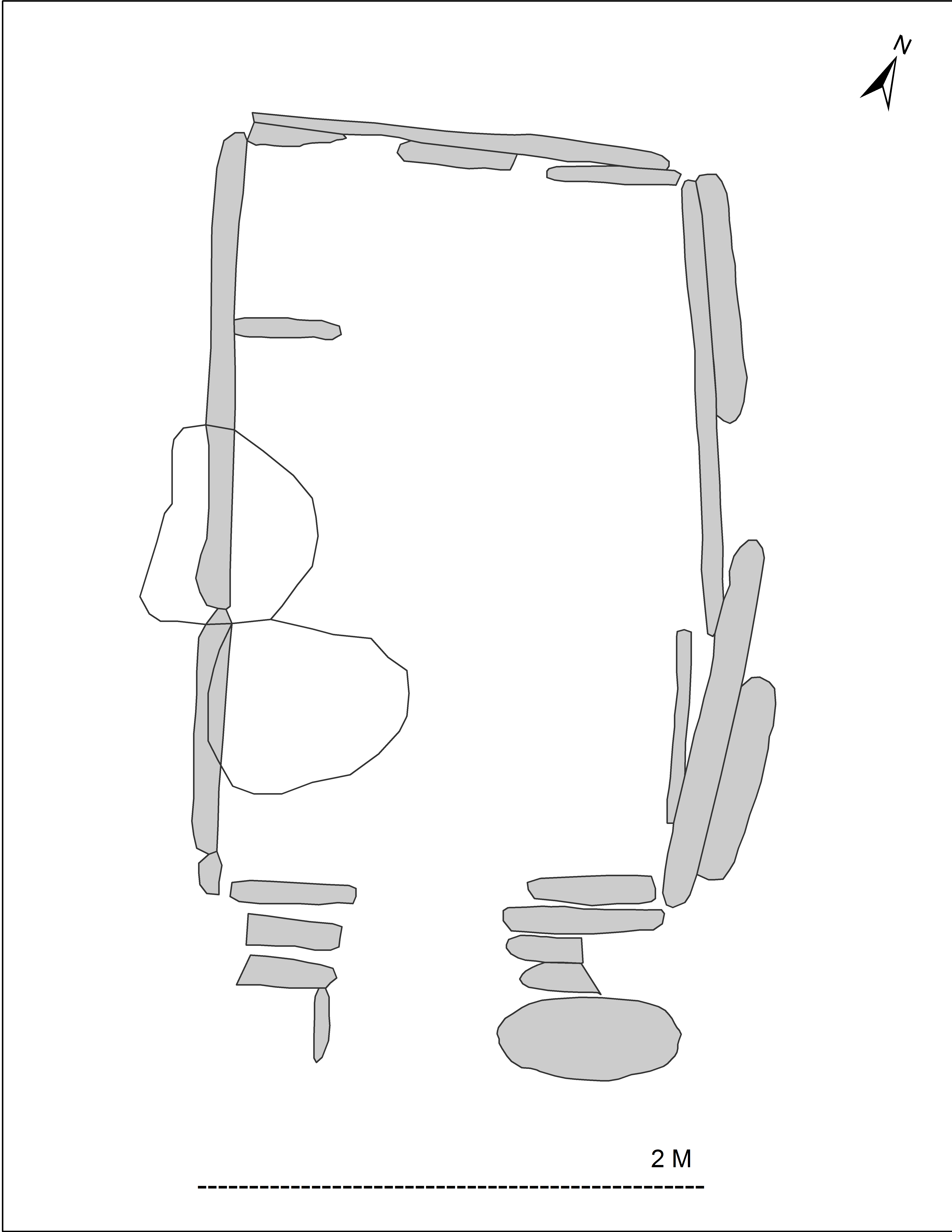

Supplement: S2 Fig — Made by Malou Blank in ArchGis 10.1, based on Sahlström [51]. (TIF) [file pone.0204662.s002.tif]

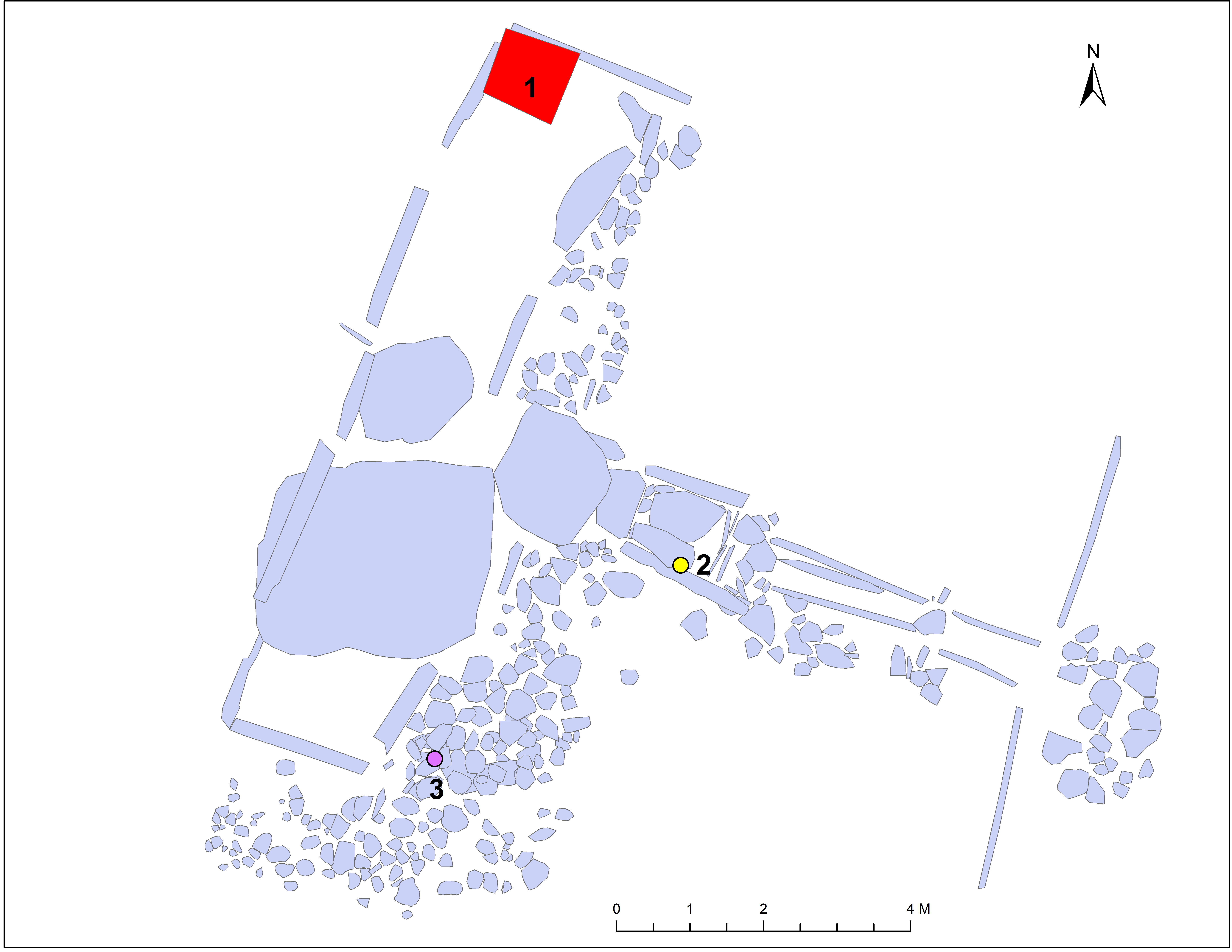

Supplement: S3 Fig — Made by Malou Blank in ArchGis 10.1. (TIF) [file pone.0204662.s003.tif]

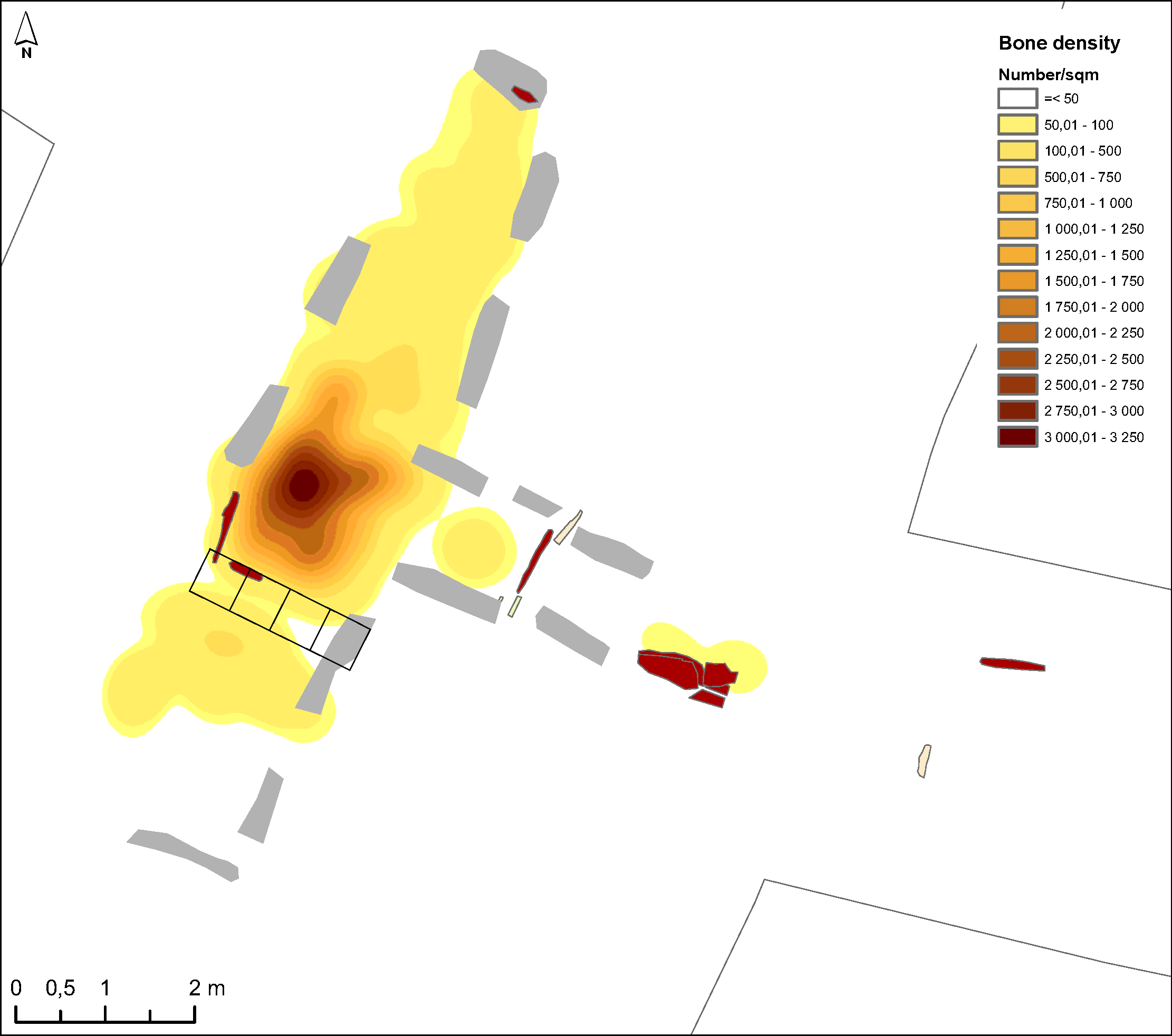

Supplement: S4 Fig — Kernel density plot, calculated from bone midpoints. Made by K-G. Sjögren in ArchGis, reprinted from Sjögren (2008) [90], under a CC BY license, with permission from University of Gothenburg, original copyright. (TIFF) [file pone.0204662.s004.tiff]
