# Supplementary material for: Dead and buried? Variation in post-mortem histories revealed through histotaphonomic characterisation of human bone from megalithic graves in Sweden
Source: PLoS One. 2018 Oct 3;13(10):e0204662. doi: 10.1371/journal.pone.0204662 (PMC6169911; doi:10.1371/journal.pone.0204662)
Supplement: S1 File — Detailed descriptions of the four graves and skeletal assemblages discussed in the article. (DOCX) [file pone.0204662.s005.docx]

# S1 File - Site descriptions

## Lilla Balltorp gallery grave, Torbjörntorp 18 (TB18)

This gallery grave was located on a ridge in the central part of Falbygden (Fig S1). The grave was dug into the ground and covered by a 0.6 m low cairn (stones mixed with soil) next to an oval flat cairn. The grave was excavated in 1948 (1) and was found to be about 7.5 meters long and 2.2 meters wide, constructed by limestone slabs and consisting of a chamber and an antechamber, divided by two slabs with a porthole. The grave filling was a mix of sand, fragmented human bones and artefacts, circa 80 cm deep, covered by a 5 cm thick layer of clean sand. Ullenius (1) noted that the shaft hole axes were heavily weathered and that most of the bone material was found close to the limestone slabs. There were no signs of later activities in the grave. However, part of the covering cairn was possibly constructed in the Iron Age, at the same time as the nearby flat cairn (2).

 Burials from the transition between Late Neolithic I and II until the end of the Early Bronze Age (period III) have been confirmed by artefact typology and new radiocarbon dates (2, 3). The human bones were commingled and fragmentary (1, 4). However, both sitting and supine positions were documented. One of the Late Neolithic individuals buried in the chamber had been unevenly cremated at about 500 °C, most probably with flesh intact (Åsa Larsson pers. comm.). The osteological material has been examined at several occasions and minimum numbers of individuals (MNI) from 37 to 77 have been suggested (4-6).  No stratigraphic information about the vertical position of the skeletal remains was recorded and therefore only the horizontal placement is known (Fig S1). Eleven individuals from different locations within the chamber were sampled for the current study (Table 1 in the main manuscript).

## Berga gallery grave, Torbjörntorp 31 (TB31)

Berga gallery grave was in excavated 1927 by Sahlström, and was later moved to a nearby location by Svensson in 1928. The gallery grave was found underneath a low cairn and the grave was constructed by limestone slabs which formed a single chamber with an antechamber (Fig S2). The grave was three by two meters large and dug into the ground. The grave was most probably covered by roof slabs (7). The chamber floor was made up by one big limestone slab and the wall slabs were set in natural cracks in the limestone bedrock (8). Inside the grave a three decimetre thick stone filling was excavated which covered a layer of black soil mixed with artefacts and human bones (7). The bones were in a fragmentary and commingled state suggesting that the buried individuals were moved to make place for new burials.  
The number of buried individuals is difficult to estimate, but apparently around 30 skulls were found in the bottom layer only (7). Considering the method of documentation used at the time of excavation, the number of buried individuals would likely have been higher. Pig phalanges were found in the grave (7), which in passage graves have been suggested to imply bodies wrapped in skins (4). When the grave material was reanalysed, a burnt slate pendant, a couple of burnt flint arrows and a piece of cremated scull were discovered, indicating that one of the individuals had been exposed to fire (Blank, ongoing work).

The cairn was probably constructed at a later phase, as an Iron Age inhumation of a child was found in the chamber (2).  Two skeletons are described as being more or less articulated and placed in a supine position with the head pointing to the north. One of them was placed with the head and upper body inside the antechamber and the rest of the body outside the grave (7). This individual, a short woman with a small shaft hole axe next to the femur, was sampled for the current study (TB31:B, Fig S2). Five more individuals from different locations and levels in the grave were also sampled and analysed (Table 1 in the main manuscript).

Human remains from both sites in Torbjörntorp are included in ongoing diet and mobility studies (9, 10) as well as aDNA research (11).

## Firse sten passage grave, Falköping Östra 1 (FÖ1)

The passage grave Firse sten underwent minor restauration in 1950 and was partly excavated in 2008 by Västergötlands museum. The chamber (8x2.5 m) and passage was constructed by limestone slabs and filled with soil, stones and human bones. The construction was surrounded by a mound, 30 m in diameter, and the chamber and most of the passage was covered by roof slabs (Fig S3). Parts of the passage of the grave had been rebuilt into a gallery grave during the Late Neolithic, and in this section at least three individuals, two adults and one child, were documented. The skeletons seemed to be articulated and one of the skeletons lay in a supine position (12). One adult from the rebuilt passage was included in this study. Furthermore, an inhumation of an adult man was found in the surrounding mound along with six bronze artefacts dated to Period IV (3, 12). The bones were commingled, probably due to secondary disturbance, as more recent remains were found in the same context. The northern part of the chamber was also excavated and in the bottom layer of the north-western corner a teenage female was placed in a contracted position on her side (12). This Middle Neolithic individual was also sampled for the current study (See Table 1 in the main manuscript and Fig S3).

Skeletal material from this grave was also included in recent mobility and subsistence studies (9, 10).

## Frälsegården passage grave, Gökhem 94 (GH94:1)

This passage grave was already destroyed when it was excavated in 1999 to 2001. Although the chamber stones had been removed and the grave ploughed over, a large amount of bone material was collected and the construction could be documented (13, 14). The grave was constructed by limestone slabs. The chamber was rectangular (ca 9x2m) with a centrally placed passage (10m long), surrounded by a mound (30 m in diameter) (Fig S4) (14).

A 20 cm thick, compact bone layer was excavated in the chamber. The bones in the upper part of the layer were more fragmented than the ones in the bottom. The minimum number of individuals (MNI) deposited in the chamber was calculated to 51, while the most likely number of individuals (MLNI) was estimated to 78. The human bones were dated to 3100-2900 cal. BC (15). During the excavation, whole and partially articulated, as well as disarticulated skeletons were documented (14, 15). The predominant position was contracted and in most cases with strongly flexed limbs. Original sitting positions have been suggested (4, 15). One individual, GH94:1, dated to the late middle Neolithic (MN B) was sampled for this study (Table 1 in main manuscript), and compared with thin sections from a previous histological study of skeletal remains from this grave (15).

The remains from this grave have been thoroughly studied, and analyses of diet, mobility, and aDNA have also been performed (4, 13-21).

## References

1. Ullenius G. 27 November, Grävningsberättelser över undersökning av en hällkista samt en kvarstående rest av ett flatröse vid Måns Larsgården, Lilla Balltorp, Torbjörntorp sn, VG, dnr: 5220. 1948.

2. Blank M. Prehistoric Activities in Megalithic Graves in Falbygden, Sweden. Lund Archaeological Review. 2016;21:49-70.

3. Blank M. Bronze Age burials in megalithic graves in Falbygden. In: Bergerbrant S, Wessmann A, editors. New Perspectives on the Bronze Age: The 13th Nordic Bronze Age Symposium; 2015 May 5; Gothenburg: Archaeopress; 2017. pp. 19-36.

4. Ahlström T. Underjordiska dödsriken. Humanosteologiska studier av neolitiska kollektivgravar. Coast to Coast-books. 18. Gothenburg: Department of Archaeology, University of Gothenburg; 2009.

5. Holmer U, Maunsback AB. Odontologische Untersuchung von Zähnen und Kiefern des Menschen aus der Steinzeit in Schweden. Odontologisk tidskrift. 1957:437-522.

6. Lennblad A. Döden i hällkistan. En material och litteraturstudie kring senneolitikums hällkistegravar. Unpublished master thesis. : University of Gothenburg; 2015.

7. Sahlström KE. Undersökning av Berga hällkista, Torbjörntorp sn, VG, dnr 3678/1927. 1927.

8. Svensson H. Berättelser förande flyttaning av hällkista samt därvid gjorda iakttagelser, Berga kalkbrott, Torbjörntorp socken, Västergötland. Antikvarisk-Topografiska Arkivet, Stockholm, Sweden. 1928.

9. Blank M. Tracing dietary change of the megalithic population in Southwestern Sweden. Megaliths, Societies, Landscapes Early Monumentality and Social Differentiation in Neolithic Europe; 16-20.06.2015; Kiel. In press.

10. Blank M, Knipper C. Neolithic mobility in western Sweden: interpretations of strontium isotope ratios of the megalithic population in Falbygden. In: Gibson C, Frieman C, Cleary K, editors. Making Journeys Archaeologies of Mobility. Oxford: Oxbow books; In press.

11. The Atlas Project; 2016. Available from: http://theatlas.se/.

12. Jankavs P. Vem var Firse? In: Alexandersson H, Andreeff A, Bünz A, editors. Med hjärta och hjärna En vänbok till professor Elisabeth Arwill-Nordbladh. GOTARC Series A Gothenburg Archaeological Studies. 5. Gothenburg: University of Gothenburg; 2014, pp. 185-198.

13. Ahlström T. Grave or ossuary? Osteological finds from a recently excavated passage tomb in Falbygden. In: Knutsson H, editor. Coast to coast - Arrival Results and Reflections Coast to Coast-books. 10. Uppsala; 2004. pp. 233-258.

14. Sjögren K-G. Fragment av ordning. Undersökning av överplöjda megalitgravar vid Frälsegården, Gökhems socken, Västergötland, 1999-2001. GOTARC Serie D nr 62, Västergötlands museum rapport 2008:23. Skara; 2008.

15. Sjögren K-G. News from Frälsegården. Aspects of Neolithic burial practices. In: Brink K, Hydén, S., Jennberg, K., Larsson, L., Olausson, D., editors. Neolithic Diversities Perspectives from a conference in Lund, Sweden. Acta Archaeologica Lundensia, Series in 8o. 65. Lund, Sweden: Department of Archaeology and Ancient History, Lund University; 2015. pp. 200-210.

16. Hinders J. Dödsrikets livshistorier. Benkemiska isotopanalyser på artikulerade och disartikulerade individer i Frälsegårdens gånggrift. Stockholm: Stockholms universitet; 2011.

17. Sjögren K-G. Modeling middle Neolithic funnel beaker diet on Falbygden, Sweden. Journal of Archaeological Science: Reports. 2017;12:295-306.

18. Sjögren K-G, Price TD. Vegetarians or meat eaters? Enamel δ13C and Neolithic diet at the Frälsegården passage tomb, central Sweden. In: Bergerbrant S, Sabatini S, editors. Counterpoint: Essays in Archaeology and Heritage Studies in Honour of Professor Kristian Kristiansen. BAR International Series. 2508. Oxford: Archaeopress; 2013. pp. 43-52.

19. Sjögren K-G, Price TD, Ahlström T. Megaliths and mobility in south-western Sweden. Investigating relationships between a local society and its neighbours using strontium isotopes. Journal of Anthropological Archaeology. 2009;28(1):85-101.

20. Skoglund P, Malmström H, Omrak A, Raghavan M, Valdiosera C, Gunther T, et al. Genomic diversity and admixture differs for Stone-Age Scandinavian foragers and farmers. Science. 2014;344(6185):747-50.

21. Skoglund P, Malmström H, Raghavan M, Stora J, Hall P, Willerslev E, et al. Origins and genetic legacy of Neolithic farmers and hunter-gatherers in Europe. Science. 2012;336(6080):466-9.
