# Supplementary material for: Dead and buried? Variation in post-mortem histories revealed through histotaphonomic characterisation of human bone from megalithic graves in Sweden
Source: PLoS One. 2018 Oct 3;13(10):e0204662. doi: 10.1371/journal.pone.0204662 (PMC6169911; doi:10.1371/journal.pone.0204662)
Supplement: S2 File — (DOCX) [file pone.0204662.s006.docx]

To the editors.

I hereby confirm that in my estimation the unevenly burnt bone, found in the bone assembly from gallery grave Torbjörntorp 18, came from an individual cremated in the flesh and where the bone itself was not subjected to temperatures exceeding 500C. This is based on the colors of the bone and the lack of fractures appearing at higher temperatures. I also agree that this information is used and referred to me.

**Åsa M Larsson**, PhD

Swedish National Heritage Board

Email: [asa.larsson@raa.se](mailto:asa.larsson@raa.se)

Tel: +46 8 5191 8241

[LinkedIn](https://se.linkedin.com/in/asamlarsson)

[Academia.edu](https://uppsala.academia.edu/AsaMLarsson)
