# Supplementary material for: Dead and buried? Variation in post-mortem histories revealed through histotaphonomic characterisation of human bone from megalithic graves in Sweden
Source: PLoS One. 2018 Oct 3;13(10):e0204662. doi: 10.1371/journal.pone.0204662 (PMC6169911; doi:10.1371/journal.pone.0204662)
Supplement: S1 Table — (DOCX) [file pone.0204662.s007.docx]

# **S1 Table – Histological data from previous study on bone from Gökhem 94:1**

| **Sample id** | **Species** | **Element** | **Level of articulation** | **OHI*** | **Pattern of bioerosion** |
| --- | --- | --- | --- | --- | --- |
| 123293 | Human | L. femur | Disarticulated | 0 | Extensive |
| 139267B | Human | L. femur | Articulated | 1 | Extensive |
| 130443G | Human | L. femur | Partially articulated | 3 | Arrested |
| 138105A | Human | R. femur | Articulated | 2 | Arrested |
| 119236 | Human | L. femur | Disarticulated | 0 | Extensive |
| 132324K | Human | L. femur | Partially articulated | 1 | Extensive |
| 136163AC | Human | L. femur | Partially articulated | 2 | Arrested |
| 134704E | Human | R. femur | Articulated | 0 | Extensive |
| 117150/115371 | Human | R. femur | Disarticulated | 0 | Extensive |
| 124039 | Human | L. femur | Disarticulated | 0 | Extensive |
| 107191 | Dog | Humerus | Articulated | 0 | Extensive |

*From unpublished report by Tom Booth, to be published in full in a forthcoming monograph: Sjögren, K-G & Ahlström, T., in press. *Anonymous Ancestors? Reconsidering burial practices in Scandinavian megalithic tombs*.
