# Supplementary material for: Dead and buried? Variation in post-mortem histories revealed through histotaphonomic characterisation of human bone from megalithic graves in Sweden
Source: PLoS One. 2018 Oct 3;13(10):e0204662. doi: 10.1371/journal.pone.0204662 (PMC6169911; doi:10.1371/journal.pone.0204662)
Supplement: S2 Table — (DOCX) [file pone.0204662.s008.docx]

# S2 Table – Additional sample information

| **Grave id** | **Location** | **Lat/Lon coordinates (WGS 84)** | **Specimen repository** | **Repository id** | **Sample id** |
| --- | --- | --- | --- | --- | --- |
| **Gökhem 94:1** | Gökhem, Falbygden, SE | Lat: 58° 9' 52,27", Long: 13° 27' 17,04" | Västergötlands museum | 18119 | GH94A, F74 |
| **Falköping Östra 1** | Falköping, Falbygden, SE | Lat: 58° 9' 10,86",  Long: 13° 34' 7,15" | Västergötlands museum | F43 | FÖ1A |
|  |  |  |  | F34 | FÖ1B |
|  |  |  |  | F40 | FÖ1C |
| **Torbjörntorp 18** | Torbjörntorp, Falbygden, SE | Lat: 58° 13' 4,65", Long: 13° 37' 15,74" | Västergötlands museum | 88966:H4 | M25 |
|  |  |  |  | 88966:S4 | M30 |
|  |  |  |  | 88966:H8 | M31 |
|  |  |  |  | 88966:S24 | M33 |
|  |  |  |  | 88966:TMG | M34 |
|  |  |  |  | 88966:TMG | M35 |
|  |  |  |  | 88966:H10-11 | M36 |
|  |  |  |  | 88966:H7 | M37 |
|  |  |  |  | 88966:H4 | M38 |
|  |  |  |  | 88966:H5 | M48 |
|  |  |  |  | 88966:S27 | M49 |
|  |  |  |  | 88966:H4d | Cat |
| **Torbjörntorp 31** | Torbjörntorp, Falbygden, SE | Lat: 58° 12' 33,10", Long: 13° 37' 57,29" | Statens historiska museum | 18522:43 | TB31:A |
|  |  |  |  | 18522:42 | TB31:B |
|  |  |  |  | 18522:43 | TB31:C |
|  |  |  |  | 18522:43 | TB31:D |
|  |  |  |  | 18522:43 | TB31:E |
|  |  |  |  | 18522:43 | TB31:F |
